# Equivalence between inverted regions of the energy gap law and inverted regions of donor–acceptor distances in photoinduced electron transfer processes in flavoproteins†

**DOI:** 10.1039/d0ra09716k

**Published:** 2021-02-26

**Authors:** Nadtanet Nunthaboot, Seiji Taniguchi, Haik Chosrowjan, Fumio Tanaka

**Affiliations:** Department of Chemistry and Center of Excellence for Innovation in Chemistry, Faculty of Science, Mahasarakham University Mahasarakham 44150 Thailand nadtanet.n@msu.ac.th; Division of Laser Biochemistry, Institute for Laser Technology Osaka 550-0004 Japan fumio.tanaka@yahoo.com

## Abstract

In the present work, we discuss about the relationship between the energy gap law and extended Dutton law in flavoproteins. The extend Dutton law is defined herein as the dependence of logarithmic rates (ln Rate) of photoinduced electron transfer (ET) from aromatic amino acids to excited isoalloxazine (Iso*) on donor–acceptor distances (Rcs). Both functions of ln Rate *vs.* negative values of the standard free energy gap and ln Rate *vs.* Rc display a parabolic behavior, when the ET rates are ultrafast. The negative values of the standard free energy gap at peaks of ln Rate [*X*_m_(ES)] were obtained for FMN-binding protein, wild-type pyranose 2-oxidase, T169S (Thr169 is replaced by Ser) pyranose 2-oxidase, and medium-chain acyl-CoA dehydrogenase. The values of Rc at peaks of ln Rate [*X*_m_(Rc)] were also obtained for these flavoproteins. The negative values of the standard free energy gap decreased with approximate linear functions of Rc. The negative values of standard free energy gap [*X*_m_(ESRc)] at Rc = *X*_m_(Rc) were evaluated using the linear functions of the negative standard free energy gap with Rc. The values of *X*_m_(ESRc) were mostly in very good agreement with the values of *X*_m_(ES). This implies that the energy gap law and the extend Dutton law are equivalent. *X*_m_(ES) values in ET donors displaying the linear extend Dutton law with Rc were obtained by energy gap law, and then *X*_m_(Rc) values were evaluated with the negative standard free energy gap. Thus, the obtained *X*_m_(Rc) values were much smaller than the Rc range obtained by the method of molecular dynamics simulation. This suggests that ET processes with linear profiles of the extend Dutton law could be parabolic when Rc becomes much shorter than the Rc range obtained by the method of molecular dynamics simulation.

## Abstract 2

Relationship between EXDL and SEGL.

## Graphical abstract

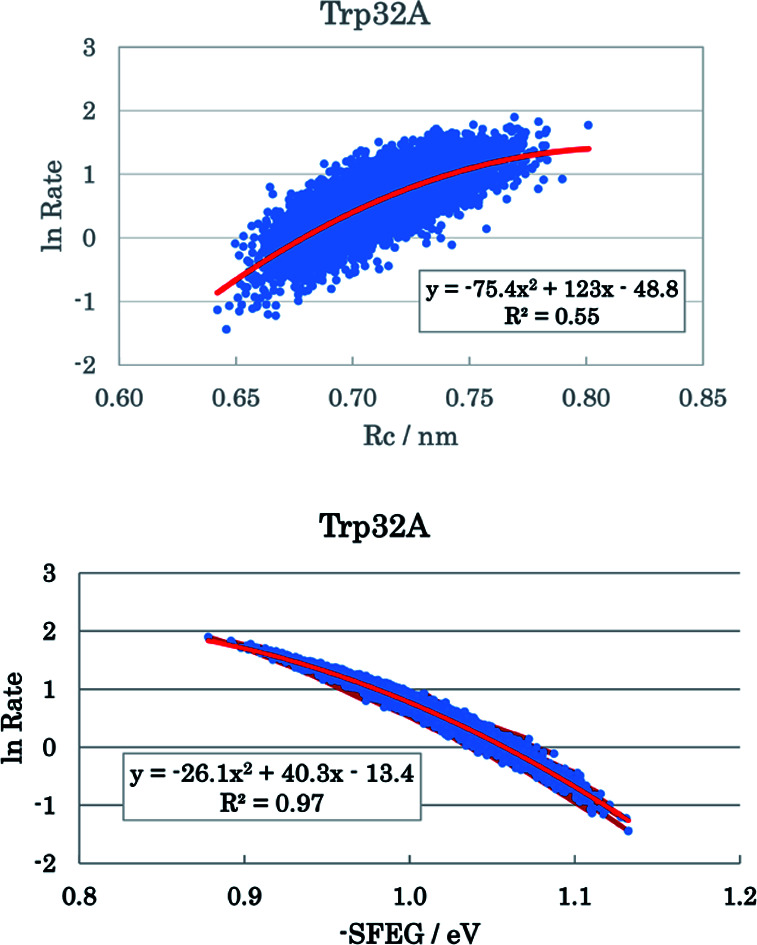

## I. Introduction

Since Marcus,^1,2^ numerous works have been reported on experimental and theoretical works of ET.^3–5^ Warshel and Parson^6^ have first introduced the method of molecular dynamics simulation (MDS) to evaluate the rate of photoinduced electron transfer (ET) in the photosynthetic reaction center of *Rb*. *sphaeroides*. Beratan *et al.*^7^ have emphasized the importance of bridges in secondary and tertiary structures in proteins.^8^ Gray and Winkler^9^ have reviewed the electron transfer phenomena in proteins. Recently, Meschi *et al.*^10^ have shown a protein network that functions effectively in a metabolic electron transfer process without specific interactions in soil bacterium *Paracoccus denitrificans*. Antonyuk *et al.*^11^ have demonstrated a direct evidence for the importance of hydrogen-bonded water at the interface in electron transfer between proteins, using the crystal structure of haem-c-Cu nitrite reductase from *Ralstonia pickettii*.

Fluorescence of flavins was first found by Weber.^12–14^ The fluorescence of riboflavin is quenched by various biological substances.^13^ Fluorescence lifetimes of flavins were first studied by means of a phase-shift method.^15,16^ The lifetimes of FAD is 2.5 ns and that of FMN 4.7 ns. The fluorescence of flavins in the solution is quenched by Trp and Tyr.^17,18^ The quenching has been demonstrated to be due to ET from Trp or Tyr to Iso*, by means of a picosecond-resolved transient absorption spectroscopy^19^ and a femtosecond-resolved transient absorption spectroscopy.^20^ In many flavoproteins, the fluorescence of flavins is remarkably quenched due to ET, and their fluorescence lifetimes become extremely short, compared to those of free flavins.^21–27^ The ET rate from the individual donor has been evaluated with an ET theory and MDS structures, using fluorescence lifetimes or decays in these flavoproteins.^24–31^

Logarithmic ET rates (ln Rates) linearly decrease with the donor–acceptor distances (Rc), known as the Dutton law.^32^ However, the ln Rate *vs.* Rc relationship often displays parabolic functions with Rc in flavoproteins, when the rates are faster than *ca.* 1 ps^−1^.^28–31^ In these donors, the values of ln Rate become smaller with Rc, which is called the inverted region of Rc (Rc-inverted region).^28–31^ In the present study, we call the ln Rate *vs.* Rc relationship the extend Dutton law (EXDL). However, it is also known that the ln Rate *vs.* standard free energy gap (SFEG) relationship displays parabolic functions, which is called the energy gap law (SEGL).^33–36^ The region of −SFEG (negative value of SFEG) greater than that at the maximum value of ln Rate is called the inverted region of SEGL (E-inverted region). It is evident that both the parabolic relationships originated from the Marcus ET theory.^1,2^

The physical picture of the Rc-inverted region is not known yet. This is physically unreasonable because the ET rate becomes slower as the Rc is shorter, despite the greater interaction energy. This phenomenon was interpreted that the Marcus theory does not hold anymore in the Rc-inverted region, and the ET phenomena should be treated by molecular orbital theory.^28,29,37,38^ Herein, we describe the relationship between EXDL and SEGL in ET processes in flavoproteins, using the Marcus–Kakitani–Mataga (MKM) theory.^1,2,39–41^

## II. Methods

### a Donor–acceptor distance

A donor–acceptor distance (Rc) is expressed as the mean distance over all pairs between aromatic atoms of donor molecules [tryptophane (Trp) or tyrosine (Tyr)] and aromatic atoms of isoallxazine (Iso) in flavoproteins. These distances were determined with the protein structures of flavoproteins obtained by methods of molecular dynamics simulation (MDS).

### b Marcus–Kakitani–Mataga ET theory

An ET reaction from an aromatic residue to Iso* is represented as follows:Iso* + Trp → Iso^−^ + Trp^+^ Δ*G*^0^

Δ*G*^0^ is the standard free energy gap (SFEG) between photo-products and reactants.

The ET rate by MKM theory is typically expressed by eqn (1):^1,2,30–32^1
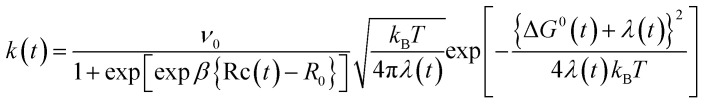


In eqn (1), *t* is the MDS time. The value of *k*(*t*) is expressed in ps^−1^.2
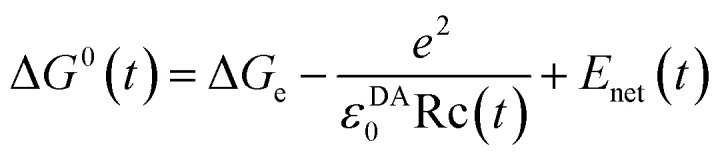


The mean value of Δ*G*^0^(*t*) over the MDS time, *t*, is Δ*G*^0^. Here Δ*G*_e_ is the electronic energy gap, obtained as follows:

3 Δ*G*_e_ = *E*_IP_ − *E*_EA_

where *E*_IP_ is the ionization potential of a donor and *E*_EA_ the electron affinity of Iso*.

The second term of eqn (2) is electrostatic (ES) energy between donor cations and acceptor anions (ESDA). *ε*^*DA*^_0_ is the static dielectric constant near donor and acceptor molecules. The third term is the ES energy between the ion-pair and ionic groups in the protein, which is described elsewhere.^28–31^

Δ*G*^0^(*t*) is dependent on the environment near a donor and an acceptor molecule as in anoxygenic photosynthetic bacteria.^42^ Tyr residues (termed YZ and YD) in Photosystem II of the bacteria display the difference in redox potential despite that they are also symmetrically arranged in the vicinity of the same electron donor (P680). The rates of electron transfer from these Tyr residues to P680 differ by at least 3 orders of magnitude. In our model, the difference in Δ*G*^0^(*t*) is taken into account in the following ways.

(a) *E*_EA_ is dependent on the environment surrounding Iso*. The hydrogen bonds between Iso and nearby amino acids should be the main factors to influence upon *E*_EA_. This quantity is determined so as to reproduce experimental fluorescence decay. The decay depends on the environment surrounding Iso and donor.

(b) *ε*^*DA*^_0_ depends on the polarity between Iso and donor molecules inside the protein.

(c) *E*_net_(*t*) is dependent on the positions of the donor and acceptor molecules.

Even though Ip values of an isolated donor molecule are used in our model, all other quantities in Δ*G*^0^(*t*) depend on the environment surrounding donor and acceptor molecules.

In eqn (1), *λ*(*t*) is the solvent reorganization energy described elsewhere,^28–31^ given by Marcus.^1,2^

The logarithmic ET rate is given by eqn (4).4
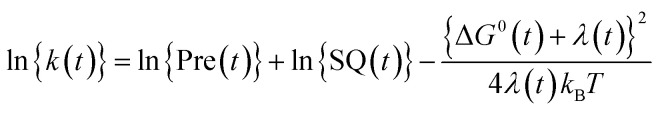
where5
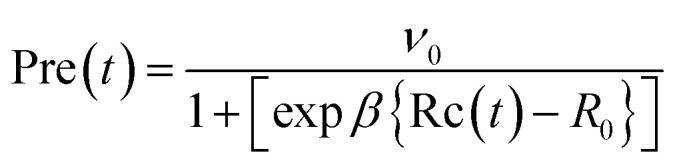
6
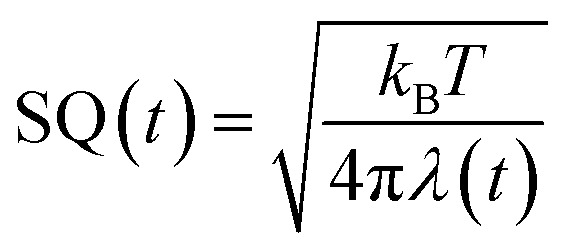


In the present work, abbreviations, ln Rate = ln{*k*(*t*)}, SFEG = Δ*G*^0^(*t*), and Rc = Rc(*t*) are used.

EXDL is expressed as the ln Rate *vs.* Rc relationship and SEGL as the ln Rate *vs.* −SFEG relationship. A relationship −SFEG *vs.* Rc is called here the ESRC.

## III. Results and discussion

### a. SEGL in FMN-binding proteins

**Fig. 1:**
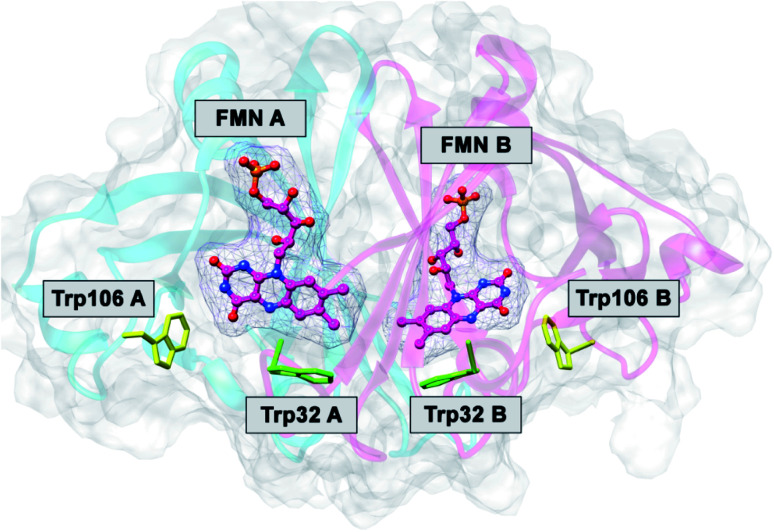
MDS structure of FBP.^29^ FMN A and FMN B denote FMN in Sub A and in Sub B. Trp32A and Trp32B denote Trp32 in Sub A and in Sub B, and so in Trp106A and Trp106B.

The FMN-binding protein (FBP) forms dimers and contains flavin mononucleotide (FMN) as a cofactor. Wild-type (WT) FBP contains Trp32 and Trp106 as ET donors. The protein structure obtained by MDS is shown in Fig. 1. The mean values of Rc over MDS time were 0.71 between Trp32 and Iso in subunit A (Sub A) and 0.68 nm in Sub B.^29^ The values of Rc were 1.03 between Trp106 and Iso in Sub A and 0.94 nm in Sub B. Parabolic behavior of EXDL in WT FBP was reported earlier.^28,29^ The approximate function of ln Rate with Rc was expressed by a parabolic function as *y* = *A*_1_*x*^2^+*B*_1_*x* + *C*_1_, where *y* = ln Rate and *x* = Rc. Fig. S1 in ESI† shows the EXDL plots of Trp32A, Trp32B, Trp106A and Trp106B.^29^ The coefficients of the parabolic functions are listed in Table S1 in ESI.† The values of Rc at maximum values of ln Rate are obtained as *X*_m_(Rc) = −*B*_1_/(2*A*_1_). The values of *X*_m_(Rc) are 0.82 in Trp32A and Trp32B, 0.90 in Trp106A and 0.94 nm in Trp106B. The range of Rc obtained by MDS are also shown in Table S1.† The range was 0.64–0.80 nm in Trp32A and 0.62–0.78 nm in Trp32B. The values of *X*_m_(Rc) in Trp32A and Trp32B are longer than these Rc range, which implies that ln Rate values in these donors are entirely in the Rc-inverted region.^29^ However, the *X*_m_(Rc) values of Trp106A and Trp106B, 0.90 and 0.94 nm, are within the Rc range 0.82–1.10 in Trp106A and 0.82–1.82 nm. Accordingly, the values of ln Rate in Trp106A and Trp106B are partly in the Rc-inverted region, and partly in the Rc-normal region.

**Fig. 2:**
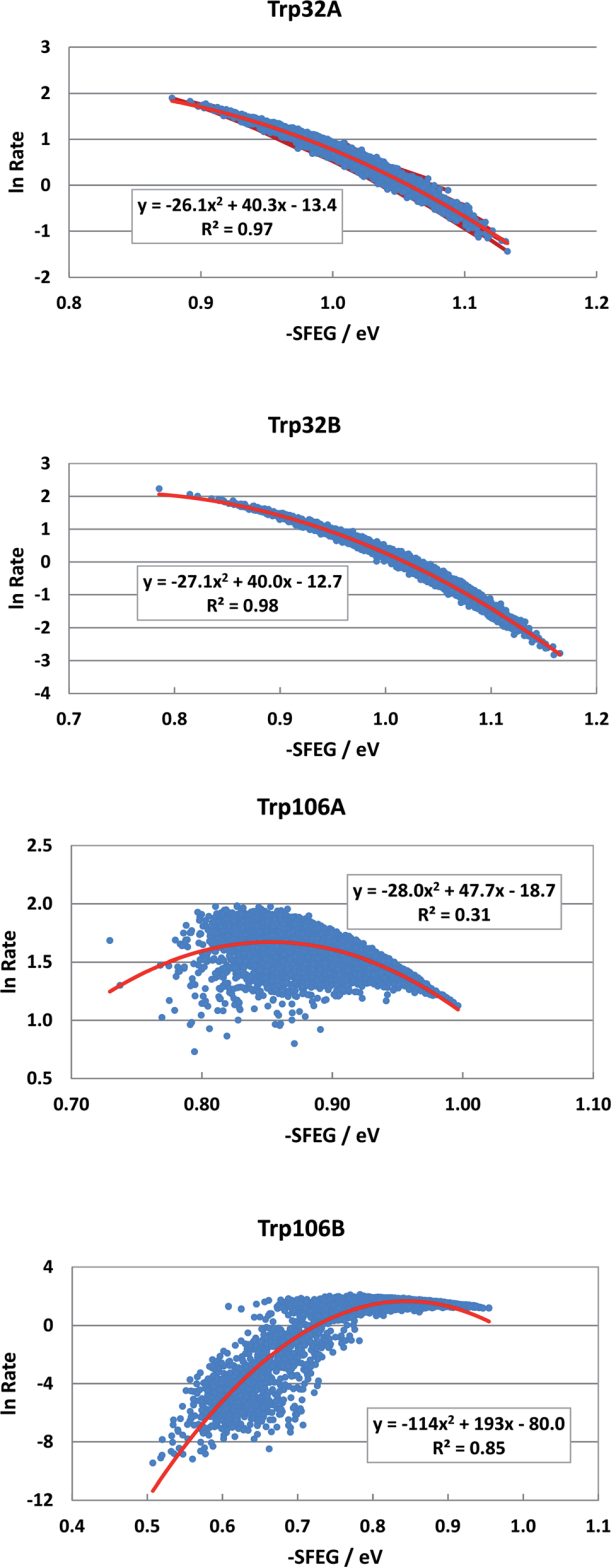
SEGL profiles in FBP. Trp32A and Trp32B denote Trp32 in Sub A and Sub B. Trp106A and Trp106B denote Trp106 in subunit A (Sub A) and in Sub B as ET donors. Insets show the approximate parabola functions of ln Rate with −SFEG. *R*^2^ denotes the determination coefficient.

Fig. 2 shows the SEGL in FBP. In any ET donor, ln Rates also depended on −SFEG with parabolic functions, as *y* = *A*_2_*x*^2^+*B*_2_*x* + *C*_2_, where *y* = ln Rate, *x* = −SFEG. The coefficients, *A*_2_, *B*_2_, and *C*_2_ are listed in Table 1. The values of *A*_2_, *B*_2_, and *C*_2_ (Trp32 in Sub A) were −26.1, 40.3 and −13.4 in Trp32A and −27.1, 40.0 and −12.7 in Trp32B. The values of *A*_2_, *B*_2_, and *C*_2_ were −28.0, 47.7 and −18.0 in Trp106A and −114, 194 and −80.0 in Trp106B.

**Table 1:** Parameters of SEGL in flavoproteins^a^

Protein	Donor^b^	Coefficient of parabolic function			*X*_m_(ES)^c^ (eV)	Range of −SFEG (eV)
		*A*_2_	*B*_2_	*C*_2_		
FBP WT	Trp32A	−26.1	40.3	−13.4	0.77	0.87–1.15
	Trp32B	−27.1	40	−12.7	0.82	0.78–1.15
	Trp106A	−28	47.7	−18	0.90	0.72–1.10
	Trp106B	−114	193	−80	0.94	0.5–0.95
P2OWT	Trp168B	−15.3	56.8	−50	1.86	1.81–1.92
	Trp168C	−13.4	50	−43.8	1.87	1.79–1.91
	Trp168D	−18.3	68.2	−60.5	1.86	1.79–1.89
P2OT169S	Trp168A	−13.4	56.4	−57	2.10	1.62–1.71
	Trp168B	−24.4	103	−1.7	2.11	1.62–1.71
	Trp168D	−22.4	95.4	−98.8	2.13	1.61–1.71
MCAD	Trp166A	−19.3	92.5	−110	2.40	2.33–2.54
	Trp166B	−9.28	43.9	−50.4	2.37	2.3–2.58
	Trp166C	−9.65	44.3	−49.1	2.30	2.25–2.41
	Trp166D	−9.5	45	−51.9	2.37	2.33–2.54

a The values of ln Rate (*y*) were plotted against −SFEG (*x*), and approximated with parabolic functions, *y* = *A*_2_*x*^2^+*B*_2_*x* + *C*_2_.

b A, B, C, D denote subunits.

c *X*_m_(ES) is *x* value at maximum in *y*, obtained with *X*_m_(ES) = −*B*_2_/(2*A*_2_).

The values of −SFEG at peak in ln Rate were obtained as *X*_m_(ES) = −*B*_2_/(2*A*_2_). These values are listed in Table 1, and were 0.77 eV in Trp32A, 0.82 eV in Trp32B, 0.90 eV in Trp106A and 0.94 eV in Trp106B. The variation ranges of −SFEG are also listed in Table 1. The values of *X*_m_(ES) were all within the range of −SFEG variation. The values of −SFEG greater than *X*_m_(ES) are called the inverted region of SEGL (E-inverted region) and those smaller than *X*_m_(ES) the E-normal region.

### b. SEGL in wild-type pyranose 2-oxidase

**Fig. 3:**
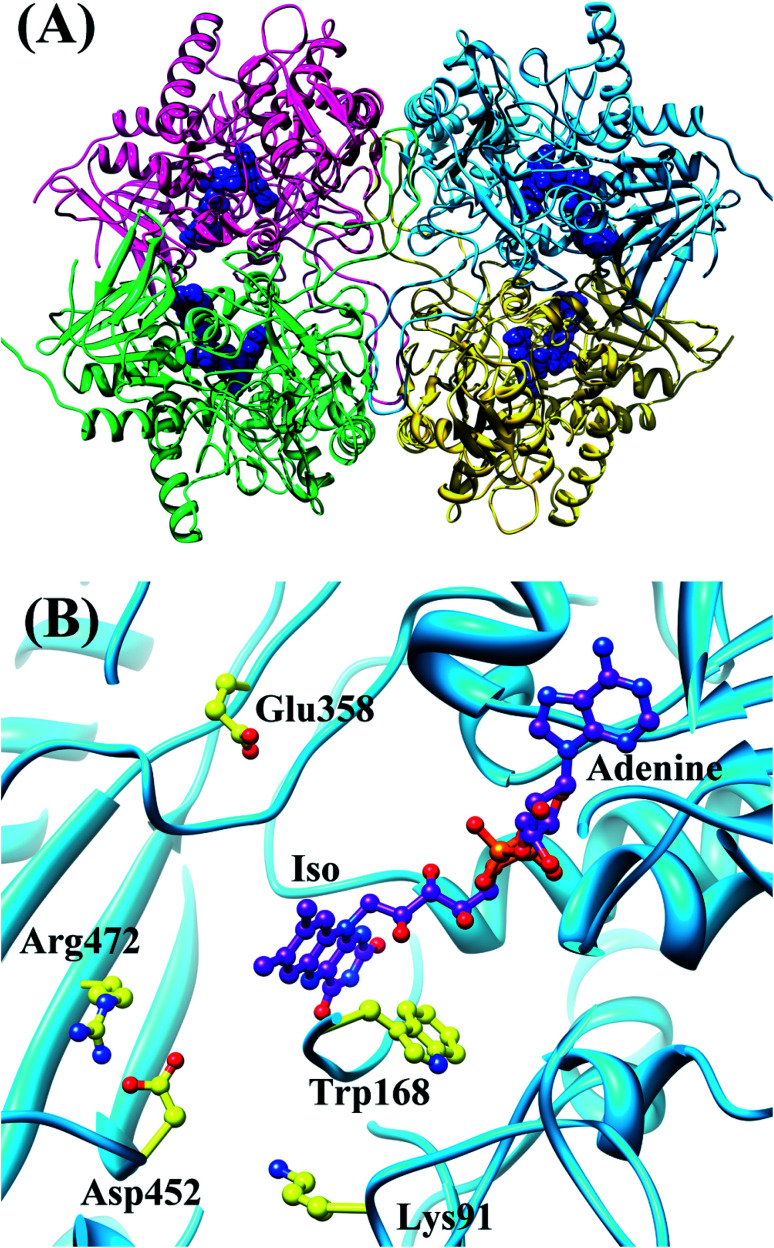
MDS structures of tetrameric WT P2O.^30^ Panel (A) shows the entire tetrameric WT P2O. The ribbons represent the protein backbones, Sub A in blue, Sub B in yellow, Sub C in pink, and Sub D in green. FAD molecules are indicated with blue stick models. Panel (B) shows the local structure at the FAD binding site in Sub A. Trp168 and nearby ionic amino acids, Glu358, Asp452, Lys91 and Arg472, are also shown in Panel (B).

Wild-type pyranose 2-oxidase (WT P2O) forms a tetramer, each subunit of which binds flavin adenine dinucleotide (FAD) as a co-factor. Fig. 3 shows the entire structure of WT P2O, and the local structure near the Iso binding site.^43^ One subunit of WT P2O contains four Trps as potential ET donors. Rc is the shortest in Trp168. The mean values of Rc between Trp168 and Iso were a little different among the four subunits, ranging from 0.73 nm (Sub B) to 0.76 nm (Sub C), whilst that for the other aromatic amino acids were all longer than 1.2 nm.^30^ The ET rate from Trp168 is the fastest among the four Trps. Furthermore, the ET rates of Trp168 in Sub B, Sub C and Sub D are much faster than that of Trp168 in Sub A, depending on the emission-wavelength.^25^ The rate is fastest at 480 nm, which is the shortest wavelength measured.^25,30^ The EXDL plots are shown in Fig. S2 in ESI.† The EXDL profiles in Sub B, Sub C and Sub D display parabolic behavior, but linear in Sub A.^30^ The coefficients of the parabolic functions are listed in Table S1 (ESI†). The values of *X*_m_(Rc) in WT P2O are 0.74 in Sub B, 0.73 in Sub C and 0.73 nm in Sub D. The range of Rc variation obtained by MDS is 0.66–0.82 in Sub B, 0.71–0.82 in Sub C and 0.69–0.83 nm in Sub D. Accordingly, the values of ln Rate are partly in the Rc-inverted region where Rcs are shorter than *X*_m_(Rc), and partly in the normal region where Rcs are longer than *X*_m_(Rc).

**Fig. 4:**
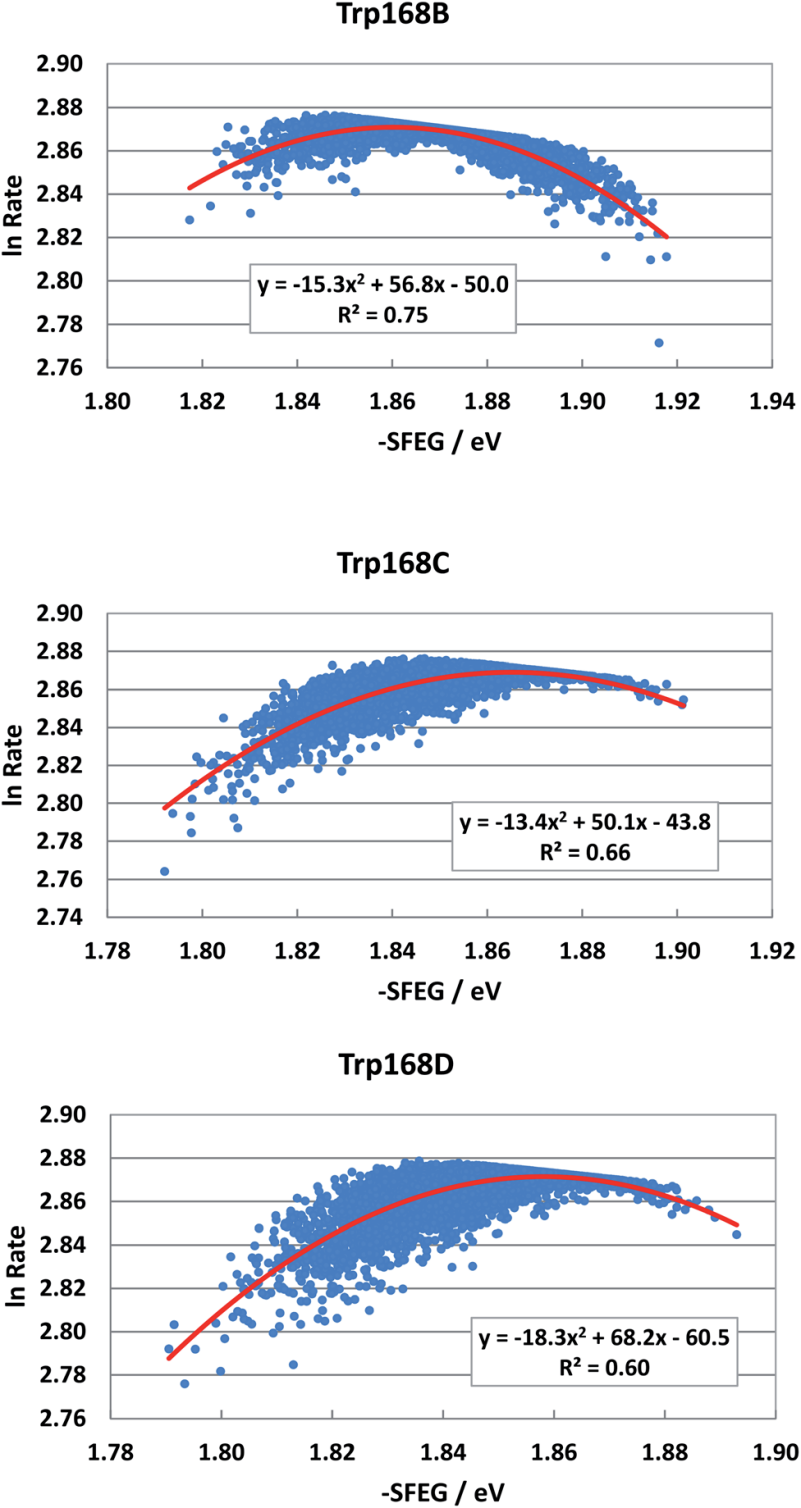
SEGL in WT P2O. Trp168B, Trp168C and Trp168D denote Trp168 in Sub B, Sub C and Sub D, respectively. These donors are fast components and emission-wavelength-dependent.^25^ The emission wavelength monitored is 480 nm. Insets show the approximate parabola functions. *R*^2^ denotes the determination coefficient.

Fig. 4 shows the SEGL plots of Trp168 at 480 nm in WT P2O. The profiles of Sub B, Sub C and Sub D were well described with parabolic functions. The coefficients are listed in Table 1. The values of *A*_2_, *B*_2_, and *C*_2_ were −15.8, 56.8 and −50.0 in Sub B, −13.4, 50.0 and −43.8 in Sub C, and −18.3, 68.2 and −60.5 in Sub D. The values of *X*_m_(ES) were 1.86 eV in Sub B, 1.87 eV in Sub C and 1.86 eV in Sub D. The range of variations in −SFEG was 1.81–1.92 eV in Sub B, 1.79–1.91 eV in Sub C and 1.79–1.89 eV in Sub D. Thus, the values of ln Rate were partly in the E-inverted region and partly in the E-normal region.

### c. SEGL in T169S pyranose 2-oxidase

**Fig. 5:**
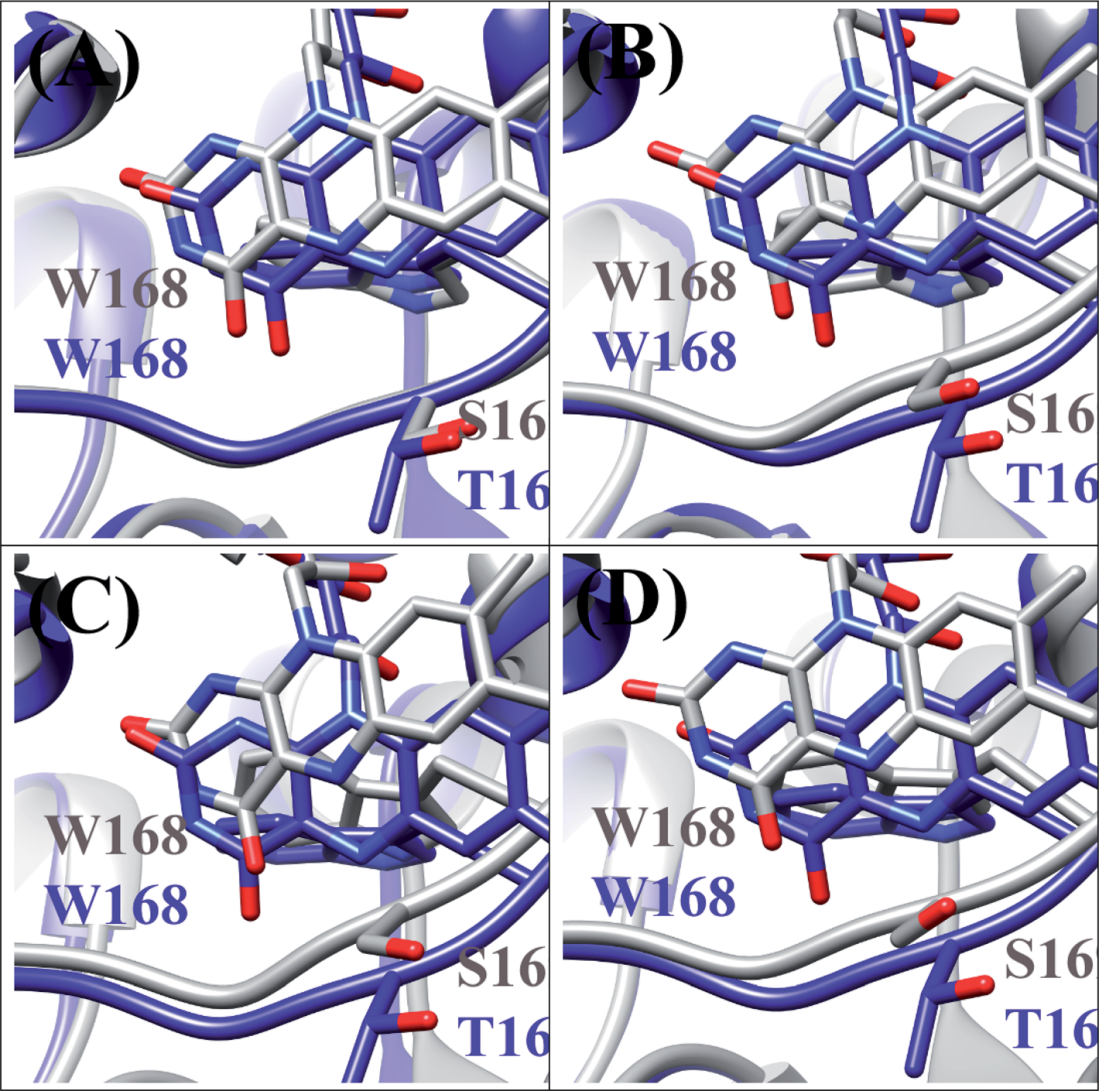
Comparison of the MDS structures at the Iso binding site between T169S P2O and WT P2O.^31,44^ The structures of T169S P2O are shown in gray and WT P2O in blue. W168 and H167 denote Trp168 and His 167, respectively, and S169 and T169 denote Ser169 and Thr169. (A), (B), (C) and (D) denote subunits.

T169S pyranose 2-oxidase (T169S P2O) is mutated P2O in which Thr169 is replaced by Ser. The mean values of Rc between Trp168 and Iso over the MDS time were a little different among the four subunits as WT P2O, ranging from 0.72 nm (Sub B) to 0.75 nm (Sub C).^44^ The protein structure of T169S P2O is compared to WT P2O, as shown in Fig. 5. The ET rate from Trp168 is also the fastest among the four Trps.^31,44^ The ET rate is dependent on the emission wavelength, fastest at 480 nm,^26^ as in WT P2O.

The EXDL plots of 480 nm in T169S P2O are shown in Fig. S3 in ESI.† The EXDL profiles in Sub A, Sub B and Sub D display a parabolic behavior, but linear in Sub C.^31^ The coefficients of the parabolic functions are listed in Table S1 (ESI†). The values of *X*_m_(Rc) in T169S P2O are 0.71 in Sub A, 0.73 in Sub B and 0.72 nm in Sub D. The range of Rc variation is 0.68–0.79 in Sub A, 0.66–0.79 in Sub C and 0.68–0.84 nm in Sub D. Thus, the values of ln Rate are partly in the Rc-inverted region and partly in the Rc-normal region.

**Fig. 6:**
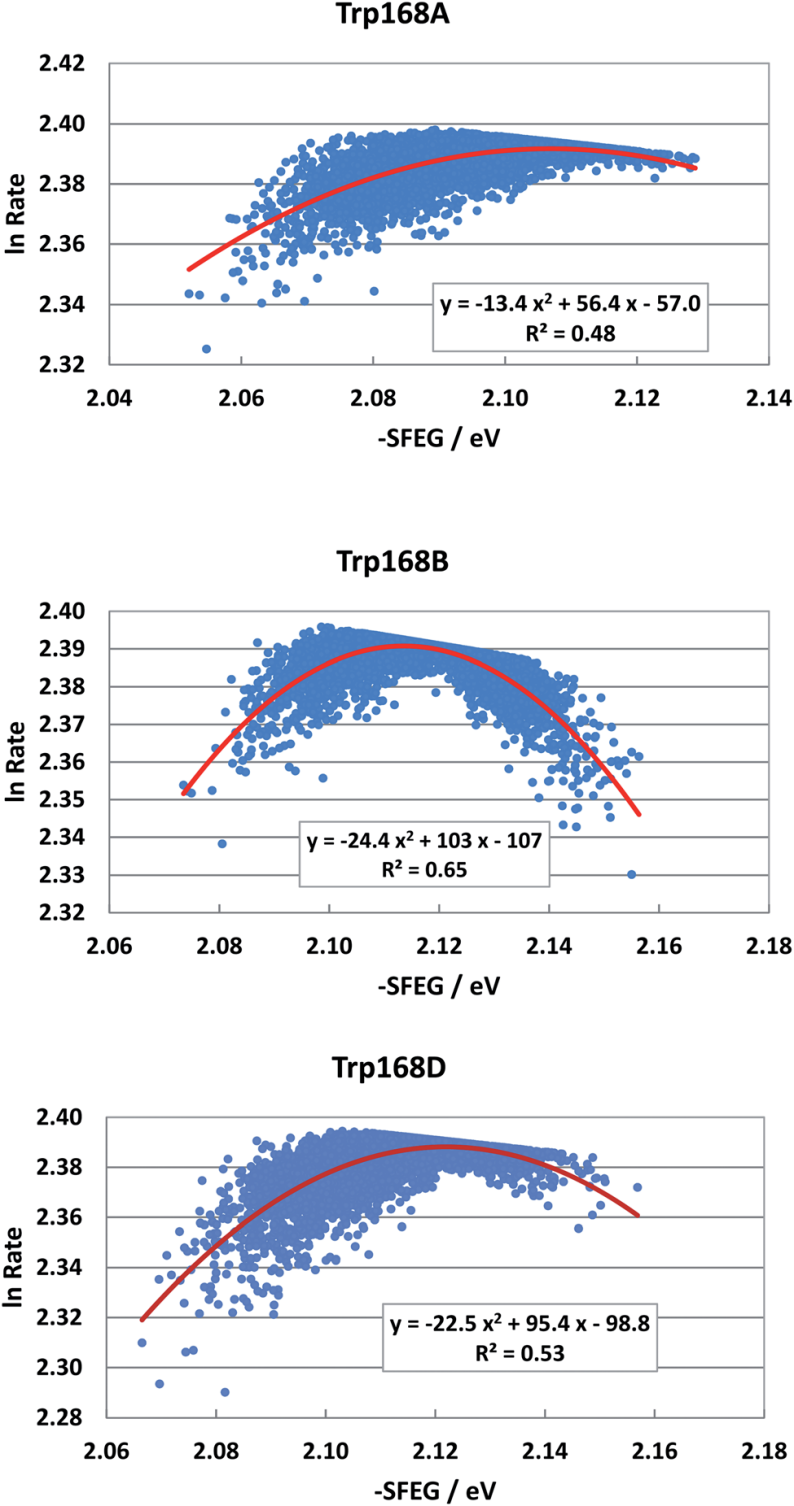
SEGL in T169S P2O. Trp168A, Trp168B and Trp168D denote Trp168 in Sub A, Sub B and Sub D, respectively. These donors are fast components and emission-wavelength-dependent for the decay measurements.^26^ The emission wavelength monitored is 480 nm. Insets show the approximate parabola functions. *R*^2^ denotes the determination coefficient.

Fig. 6 shows the SEGL plots of Trp168 at 480 nm in T169S P2O. The profiles of Sub A, Sub B and Sub D were well described with parabolic functions as in WT P2O. The coefficients are listed in Table 1. The values of *A*_2_, *B*_2_, and *C*_2_ were −13.4, 56.4 and −57.0 in Sub A, −24.4, 103 and −1.7 in Sub B, and −22.4, 95.4 and −98.8 in Sub D. The values of *X*_m_(ES) were 2.10 eV in Sub A, 2.11 eV in Sub B and 2.13 eV in Sub D. The range of variations in −SFEG was 1.62–1.71 eV in Sub A, 1.62–1.71 eV in Sub B and 1.61–1.71 eV in Sub D. Thus, the values of ln Rate were partly in the E-inverted region, and partly in the E-normal region.

### d. SEGL in medium-chain acyls-CoA dehydrogenase

**Fig. 7:**
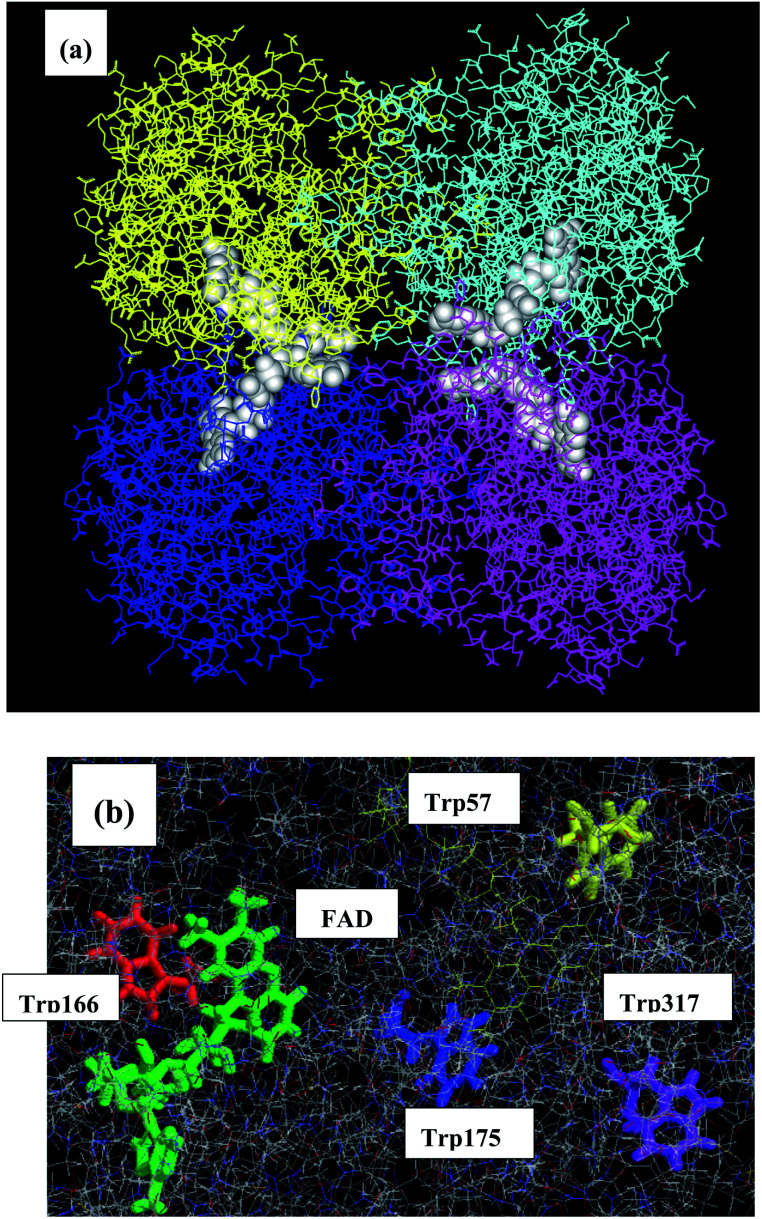
MDS structures of MCAD^45,46^ Panel (a) shows the entire structure of the tetramer, and panel (b) shows the local structure near the FAD-binding site. MCAD contains four Trps as potential donors. Trp166 is shown in green, Trp175 in red, Trp57 in blue and Trp317 in yellow. The structures of lower panel are prepared with RasWin Molecular Graphics (http://www:Rasmol.org).

Medium-chain acyls-CoA dehydrogenase (MCAD) forms a tetramer and binds one mole of FAD per subunit as in P2Os. The MDS structure is shown in Fig. 7. The mean values of Rc between Iso and Trp166 over all snapshots were shortest among the donors: 0.72 in Sub A, 0.75 in Sub B, 0.73 in Sub C, and 0.75 nm in Sub D.^45,46^ The distances of other Trps were much longer than those of Trp166. The ET rate from Trp166 is the fastest among the four Trps.^45,46^

The EXDL plots are shown in Fig. S4 in ESI.†^45^ The EXDL profiles all in Sub A, Sub B, Sub C and Sub D display a parabolic behavior. The coefficients of the parabolic functions are listed in Table S1 (ESI†). The values of *X*_m_(Rc) in Trp166 are 0.76 in Sub A, 0.81 in Sub B, 0.74 in Sub C and 0.80 nm in Sub D. The range of Rc variation is 0.77–1.0 in Sub A, 0.72–0.97 in Sub B, 0.75–0.95 in Sub C, and 0.75–1.0 nm in Sub D.^45^ The values of ln Rate are partly in the Rc-inverted region and partly in the Rc-normal region.

**Fig. 8:**
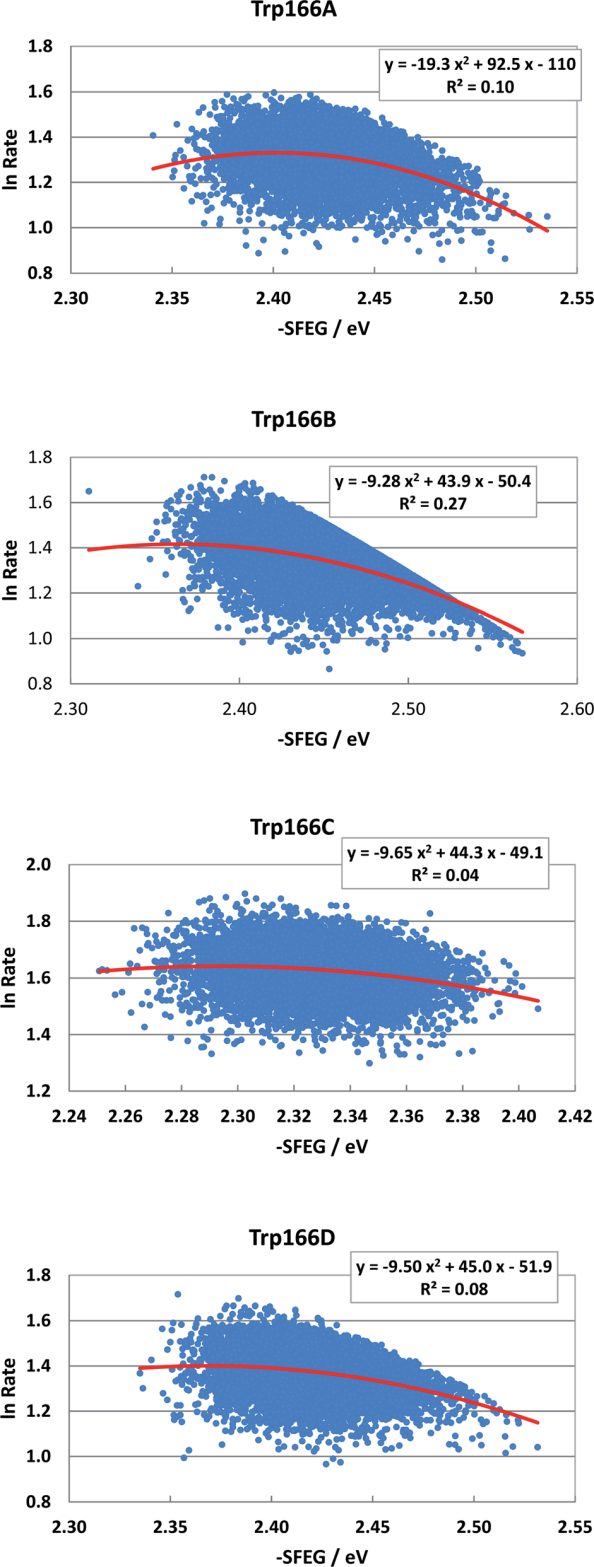
SEGL in MCAD. Trp166A, Trp166B, Trp166C and Trp166D denote Trp166 in Sub A, Sub B, Sub C and Sub D, respectively. The values of ln Rate are taken from the work reported.^45^ Insets show the approximate parabola functions. *R*^2^ denotes the determination coefficient.

Fig. 8 shows the SEGL plots of Trp166 in MCAD. The values of ln Rate are taken from the work reported.^46^ The profiles in all subunits were well described with parabolic functions. The coefficients were listed in Table 1. The values of *A*_2_, *B*_2_, and *C*_2_ were obtained as −19.3, 92.5 and −110 in Sub A, −9.28, 49.3 in Sub C and −50.4 in Sub B, and −9.65, 44.3 and −49.1 in Sub C, and −9.5, 45.0 in Sub C and −51.9 in Sub D. The values of *X*_m_(ES) were 2.40 eV in Sub A, 2.37 eV in Sub B, 2.30 eV in Sub C and 2.37 eV in Sub D. The range of variations in −SFEG was 2.33–2.54 eV in Sub A, 2.3–2.58 eV in Sub B, 2.25–2.41 eV in Sub C, and 2.33–2.54 eV in Sub D. Thus, the values of ln Rate were partly in the E-inverted region and partly in the E-normal region, in all subunits.

### e. −SFEG *vs.* Rc relationship

**Fig. 9:**
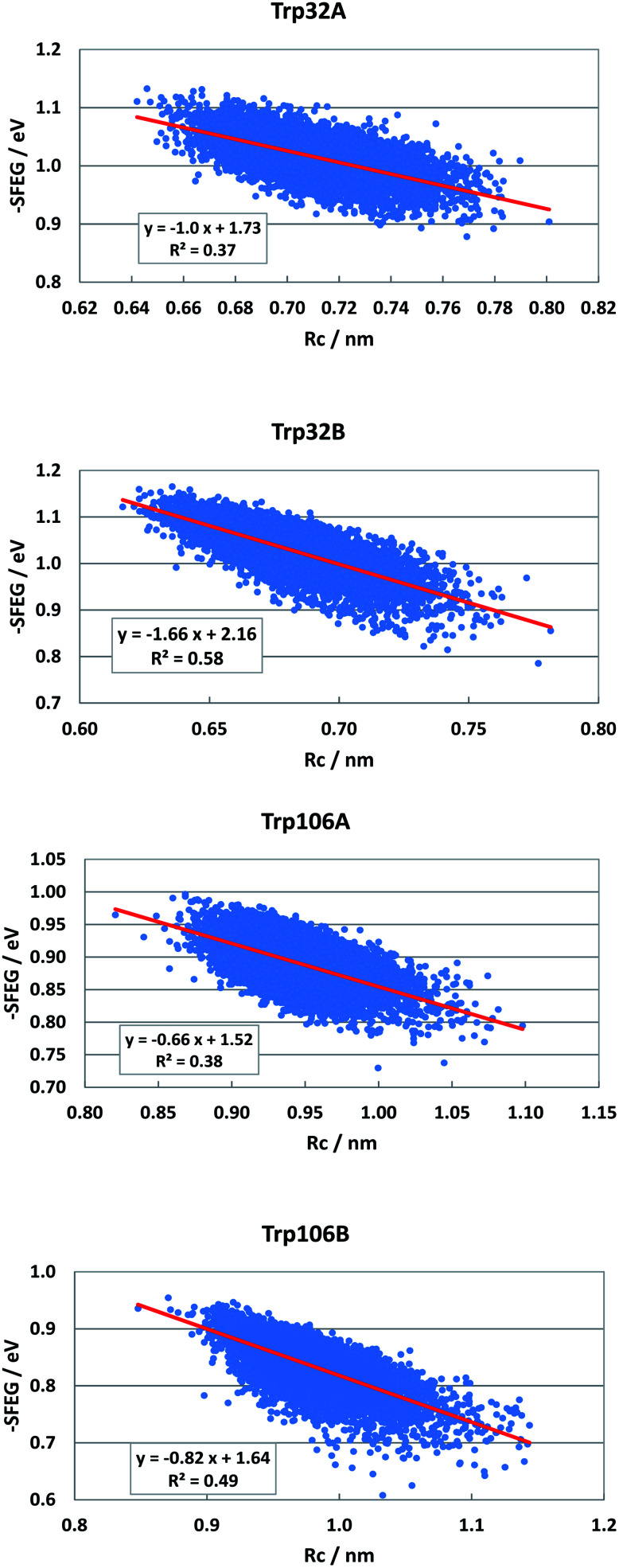
ESRC profiles in FBP. Trp106 in Sub B has two conformations with Rc shorter than 1.15 nm and longer than 1.15 nm.^29^ ESRC profile of Trp106B is shown with Rc shorter than 1.15 nm.

Dependencies of −SFEG on Rc were examined on the above-mentioned flavoproteins. The relationship is called here ESRC. Fig. 9 shows the profile of ESRC in FBP. The values of −SFEG decreased with Rc in all Trp32A, Trp32B, Trp106A and Trp106B. The relation was approximated by a linear function of *y* = *B*_3_*x* + *C*_3_, where *y* = −SFEG, *x* = Rc. The coefficients *B*_3_ and *C*_3_ are listed in Table 2. The values of *B*_3_ and *C*_3_ were −1.0 and 1.73 in Trp32A, −1.66 and 2.16 in Trp32B, −0.66 and 1.52 in Trp106A, and −0.82 and 1.64 in Trp106B.

**Table 2:** Parameters of ESRC^a^

Protein	Donor^b^	Coefficients of linear function		*X*_m_(Rc)^c^ (nm)	*X*_m_(ES)^d^ (eV)	*X*_m_(ESRc)^e^ (eV)
		*B*_3_	*C*_3_			
FBP WT	Trp32A	−1	1.73	0.82	0.77	0.91
	Trp32B	−1.66	2.16	0.82	0.82	0.80
	Trp106A	−0.66	1.52	0.90	0.90	0.92
	Trp106B	−0.82	1.64	0.94	0.94	0.87
P2OWT	Trp168B	−0.58	2.3	0.74	1.86	1.87
	Trp168C	−0.6	2.3	0.73	1.87	1.86
	Trp168D	−0.44	2.17	0.73	1.86	1.85
P2OT169S	Trp168A	−0.48	2.44	0.71	2.10	2.10
	Trp168B	−0.51	2.48	0.73	2.11	2.11
	Trp168D	−0.39	2.4	0.72	2.13	2.12
MCAD	Trp166A	−0.32	2.71	0.76	2.40	2.47
	Trp166B	−0.45	2.83	0.81	2.37	2.46
	Trp166C	−0.34	2.62	0.74	2.30	2.37
	Trp166D	−0.38	2.75	0.80	2.37	2.45

a Relationship, Rc *vs.* −SFEG. The values of −SFEG were approximated as *y* = *B*_3_*x* + *C*_3_, where *y* = −SFEG, and *x* = Rc.

b A, B, C, D denote subunits.

c *X*_m_(Rc) = −*B*_1_/(2*A*_1_). The values *X*_m_(Rc) are also listed in Table S1 in ESI.

d *X*_m_(ES) = −*B*_2_/(2*A*_2_). The values of *X*_m_(ES) are also listed in Table 1.

e *X*_m_(ESRc) = *B*_3_ *X*_m_(Rc) + *C*_3_. The *X*_m_(ESRc) implies *X*_m_(ES) value obtained with ESRC relationship.

**Fig. 10:**
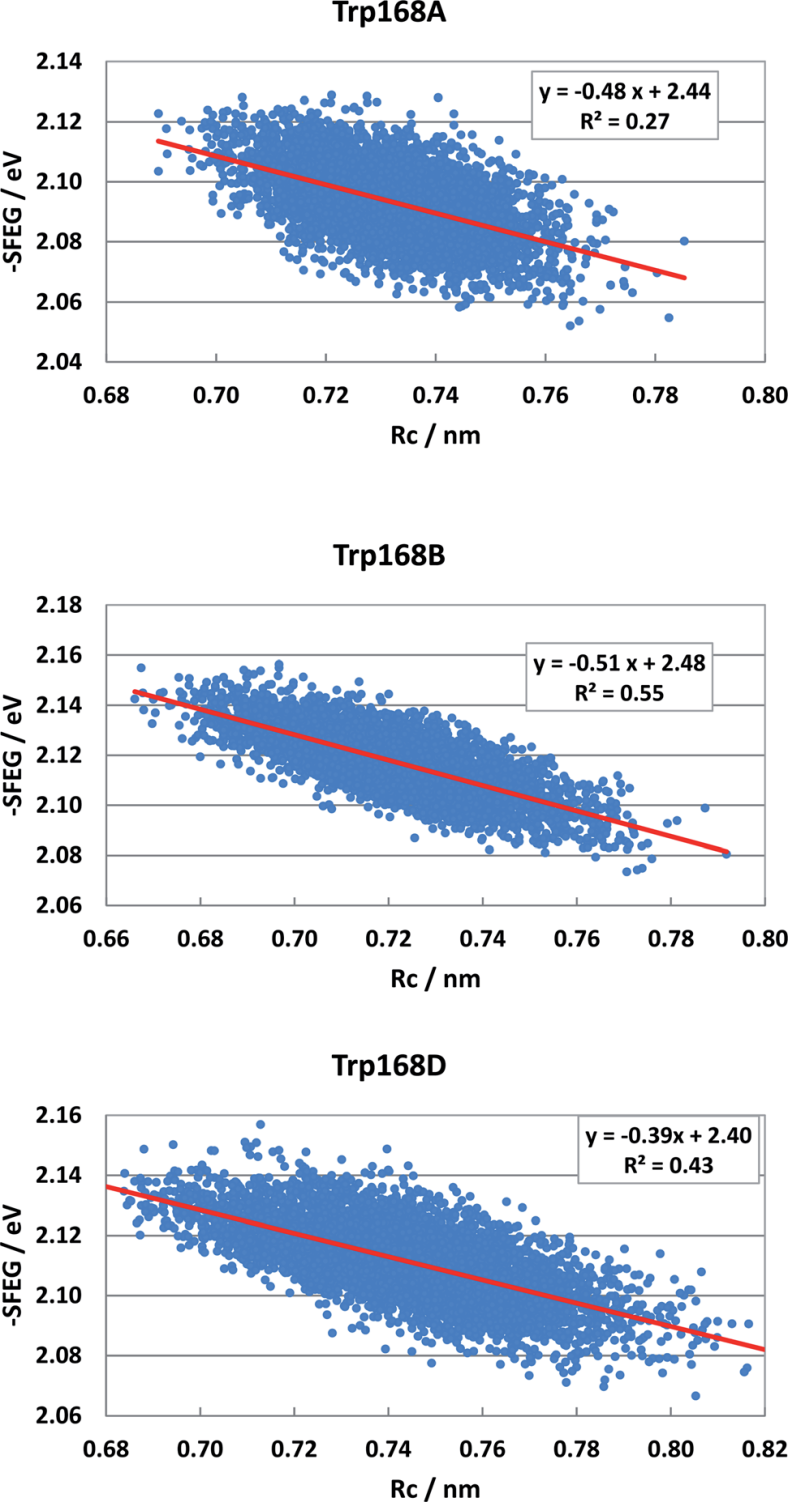
ESRC profiles in T169S P2O. Trp168A, Trp168B, and Trp168D are fast components and dependent on the emission wavelength for the decay measurements. The emission wavelength is 480 nm.^26^ Insets show the approximate linear functions. *R*^2^ denotes the determination coefficient. The values of SFEG and Rc are taken from ref. 31.

Fig. S5 (SI†) shows the ESRC in WT P2O. The values of −SFEG also decreased with Rc in WT P2O. The values of *B*_3_ and *C*_3_ were −0.581 and2.3 in Sub B, −0.60 and 2.30 in Sub C, and −0.44 and 2.17 in Sub D. Fig. 10 shows the ESRC in T169S P2O. The values of *B*_3_ and *C*_3_ were −0.48 and 2.44 in Sub A, −0.51 and 2.48 in Sub B, and −0.39 and 2.40 in Sub D. Fig. S6 (ESI†) shows the ESRC in MCAD. The values of *B*_3_ and *C*_3_ were −0.32 and 2.71 in Sub A, −0.45 and 2.83 in Sub B, −0.34 and 2.62 in Sub C, and −0.38 and 2.75 in Sub D. Sometimes, the linearity between −SFEG and Rc was not good.

### f. Comparison between *X*_m_(ES) values obtained by SEGL and *X*_m_ values obtained by EXDL and ESRC

The values of *X*_m_(ES) obtained by SEGL are shown in Table 1 as stated above. The values of *X*_m_(Rc) are listed in Table S1 in ESI† as discussed above. The relationship between −SFEG and Rc was obtained by ESRC. A value of −SFEG at Rc = *X*_m_(ES) was obtained with ESRC as *X*_m_(ESRc) = *B*_3_ *X*_m_(Rc) + *C*_3_. The values of *X*_m_(ESRc) in the eV unit are listed in Table 2. The values of *X*_m_(ESRc) in FBP were 0.91 in Trp32A, 0.80 eV in Trp32B, 0.92 eV in Trp106A and 0.87 eV in Trp106B. The values of *X*_m_(ES) obtained by SEGL were 0.77 eV in Trp32A, 0.82 eV in Trp32B, 0.90 eV in Trp106A and 0.94 eV in Trp106B (see Table 1). The values of *X*_m_(ESRc) were quite close to those of *X*_m_(ES), except for Trp32A, in which the values of *X*_m_(ESRc) and *X*_m_(ES) were 0.91 and 0.77 eV. In WT P2O, the values of *X*_m_(ESRc) were 1.87 eV in Trp168B, 1.86 eV in Trp168C, and 1.85 eV in Trp168D. The values of *X*_m_(ES) were 1.86 eV in Trp168B, 1.87 eV in Trp168C and 1.86 eV in Trp168D. The values of *X*_m_(ESRc) and *X*_m_(ES) were almost identical in any donor. In T168S P2O, the values of *X*_m_(ESRc) were 2.10 eV in Trp168A, 2.11 eV in Trp168B, and 2.12 eV in Trp168D. The values of *X*_m_(ES) were 2.10 eV in Trp168A, 2.11 eV in Trp168B and 2.13 eV in Trp168D. The values of *X*_m_(ESRc) and *X*_m_(ES) were also almost identical in any donor. In MCAD, the values of *X*_m_(ESRc) were 2.47 eV in Trp166A, 2.46 eV in Trp166B, 2.37 eV in Trp166C and 2.45 eV in Trp166D. The values of *X*_m_(ES) were 2.40 eV in Trp166A, 2.37 eV in Trp166B, 2.30 eV in Trp166C and 2.37 eV in Trp166D. The values of *X*_m_(ESRc) in any donor agreed very well with those of *X*_m_(ES).

Thus, the values of *X*_m_(ES) agreed well with those of *X*_m_(ESRc) in donors, ln Rates of which display a parabolic behavior in EXDL.

### g. SEGL in ET donors with linear behavior of EXDL

**Fig. 11:**
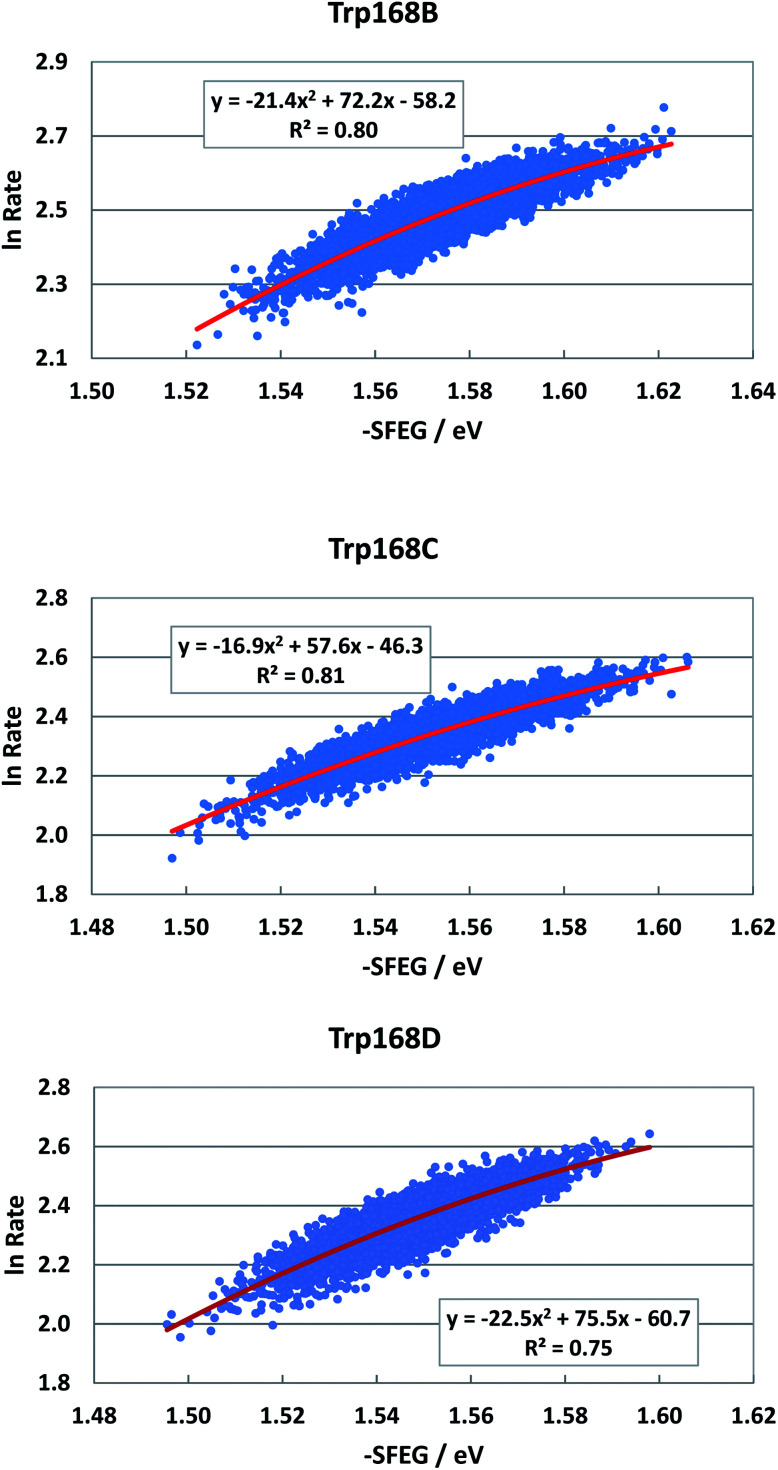
SEGL in WT P2O in the normal region. The emission wavelength monitored for the decay measurements is 580 nm.^25^ The values of SFEG and ET rates are taken from ref. 30.

EXDL profiles display linear functions of Rc when ET rates are relatively slow.^32^ Fig. S7 in ESI† shows the EXDL profiles of ET processes in the fast component of Trp168 at 580 nm and in the slow component of Sub A in WT P2O.^30^ Coefficients of the linear functions of EXDL, *y* = *B*_5_*x* + *C*_5_, are listed in Table 3. The values of coefficients, *B*_5_ and *C*_5_, are −4.61 and 5.87 in Sub B, −5.11 and 6.22 in Sub C, and −4.61 and 5.81 in Sub D. The coefficients in the slow component of Sub A were −18.3 and 7.75.^30^ The SEGL profiles in WT P2O are shown in Fig. 11. The SEGL profiles displayed all approximate parabolic functions, *y* = *A*_4_*x*^2^+*B*_4_*x* + *C*. The values of coefficients in WT P2O, *A*_4_, *B*_4_ and *C*_4_, were −21.4, 72.2 and −58.2 in Trp168B, −16.9, 57.6, and −46.3 in Trp168C, and −22.5, 75.5, and −50.7 in Trp168D. The values of *X*_m_(ES) were 1.69 eV in Trp168A, 1.70 eV in Trp168B and 1.68 eV in Trp168D.

**Table 3:** Relationship among ln Rate, −SFEG and Rc in ET processes with linear EXDL^a^

Protein	Donor^b^	Wave-length (nm)	SEGL^c^			*X*_m_(ES)^f^ (eV)	EXDL^d^		*X*_m_(Rc)^g^ (nm)	ESRC^e^	
			*A*_4_	*B*_4_	*C*_4_		*B*_5_	*C*_5_		*B*_6_	*C*_6_
WT P2O	Trp168B	580	−21.4	72.2	−58.2	1.69	−4.61	5.87	0.54	−0.581	2.00
	Trp168C	580	−16.9	57.6	−46.3	1.70	−5.11	6.22	0.51	−0.6	2.01
	Trp168D	580	−22.5	75.5	−50.7	1.68	−4.61	5.81	0.46	−0.441	1.88
T169S P2O	Trp168A	580	−10.8	42.3	−39.1	1.96	−6.78	6.34	0.07	−0.476	1.99
	Trp168B	580	−15.7	61.3	−57	1.95	−6.61	6.3	0.15	−0.508	2.03
	Trp168D	580	−25.8	94.9	−85.1	1.84	−6.15	5.98	0.29	−0.388	1.95

a In these ET systems the ln Rate linearly decreases with Rc.

b A, B, C, and D denote subunits.

c SEGL was approximated by a parabolic function, *y* = *A*_4_*x*^2^+*B*_4_*x* + *C*_4_, where *y* = ln Rate, *x* = −SFEG.

d EXDL was approximated by a linear function, *y* = *B*_5_*x* + *C*_5_.

e ESRC was approximated by a linear function, *y* = *B*_6_*x* + *C*_6_.

f The value of −SFEG with maximum value in ln Rate.

g The value of Rc (*x*) obtained by the equation, *y* = *B*_6_*x* + *C*_6_, where *y* = *X*_m_(ES).

**Fig. 12:**
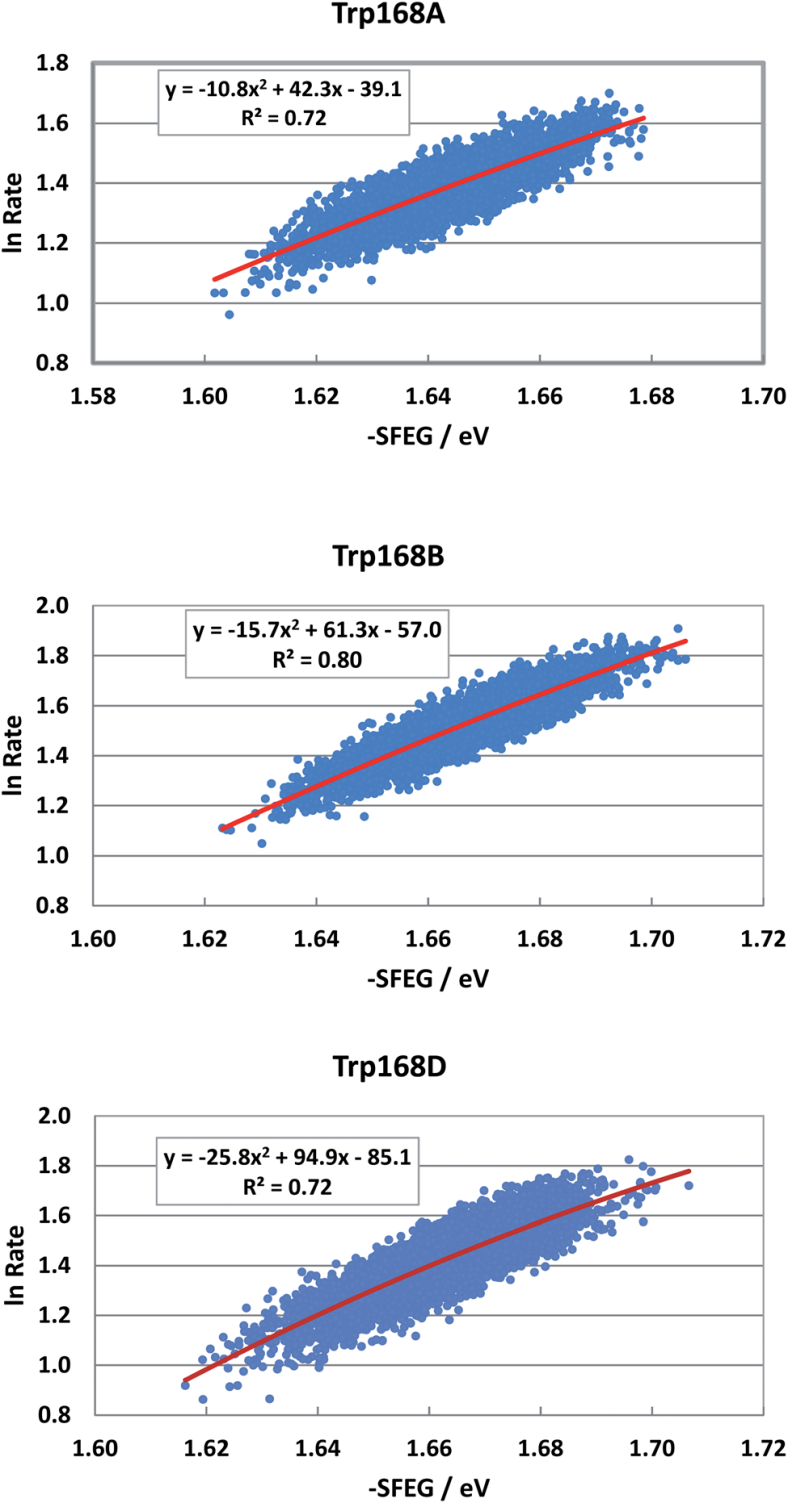
SEGL in T169S P2O in the normal region of −SFEG. The emission wavelength monitored for the decay measurements is 580 nm.^25^ The values of ET rates and SFEG are taken from ref. 31.

Fig. S8 in ESI† shows the EXDL profiles of the donors of fast components at 580 nm of emission wavelength, and of slow component of Sub C in T169S P2O.^31^ The EXDL profiles display approximate linear functions. The coefficients of the linear functions of EXDL, *y* = *B*_5_*x* + *C*_5_, are listed in Table 3. The values of coefficients, *B*_5_ and *C*_5_, are −6.78 and 6.34 in Sub A, −6.61 and 6.3 in Sub B, and −6.15 and 5.98 in Sub D. The coefficients in the slow component of Sub C were −17.3 and 10.2.^31^ The SEGL profiles in T169S P2O are shown in Fig. 12. The SEGL profiles displayed all approximate parabolic functions, *y* = *A*_4_*x*^2^+*B*_4_*x* + *C*. The values of coefficients in T169S P2O, *A*_4_, *B*_4_ and *C*_4_, were −10.8, 42.3 and −39.1 in Trp168A, −15.7, 63.1, and −57 in Trp168B, and −25.8, 94.9, and −85.1 in Trp168D. The values of *X*_m_(ES) were 1.96 eV in Trp168A, 1.95 eV in Trp168B and 1.84 eV in Trp168D.

The values of −SFEG were approximated to be linear functions of Rc, as mentioned above: *y* = *B*_6_*x* + *C*_6_, where *x* = Rc and *y* = −SFEG. The values of the coefficients, *B*_6_ and *C*_6_, are listed in Table 3. In WT P2O, the values of *B*_6_ and *C*_6_ were −0.581 and 2.00 in Trp168B, −0.60 and 2.01 in Trp168C, and −0.441 and 1.88 in Trp168D. In T169S P2O, the values of *B*_6_ and *C*_6_ were −0.476 and 1.99 in Trp168A, −0.508 and 2.03 in Trp168B, and −0.388 and 1.95 in Trp168D. The values of *X*_m_(Rc) were evaluated with the linear relationship of *y* = *B*_6_*x* + *C*_6_, where *y* = *X*_m_(ES) and *x* = *X*_m_(Rc). The values of *X*_m_(Rc) are listed in Table 3. The values of *X*_m_(Rc) were 0.54 in Trp168B, 0.51 in Trp168C, and 0.46 nm in Trp168D in WT P2O. The values of *X*_m_(Rc) were 0.07 in Trp168A, 0.15 in Trp168B, and 0.29 nm in Trp168D in T169S P2O. These values of *X*_m_(Rc) were much smaller than the range of Rc variation listed in Table S1† in ESI. Thus, the parabolic behavior in EXDL could be obtained when Rc becomes shorter around *X*_m_(Rc) in ET donors, the logarithmic ET rates of which display linear functions with Rc in the range of Rc obtained by MDS structures.

The origin of the parabolic behavior of SEGL is rather simple, because −SFEG contains only in {Δ*G*^0^(*t*)+*λ*(*t*)}^2^ term in eqn (4). The dependence of ln Rate on Rc, however, is not so simple, because many terms in eqn (1) or (4) depend on Rc, as shown in the time-dependent terms. The behavior of EXDL with Rc may be classified into three groups from the point of view of the approximate functions, (a) parabolic function, (b) linear function, and (c) no clear functional relation. Linear profiles of EXDL along Rc are found in WT P2O^30^ and T169S P2O^31^ at emission wavelengths other than 480 nm, and slow components of Sub A in WT P2O^30^ and of Sub C in T169 P2O,^31^ as described above, and in d-amino acid oxidase.^47^ Sometimes EXDL profiles do not display any clear relationship between ln Rate and Rc.^48^

The Tyr cation radical plays a very important role in photosystem II to produce molecular oxygens.^49,50^ ET from Tyr in flavin photoreceptors is also essential for biological functions of AppA^51^ and TePixD.^52^ Tyr residues in flavodoxins from *Desulfovibrio vulgaris*^53^ and *Helicobacter pylori*^48^ are donors which display ultrafast ET to Iso*. Normally, the ET rates from Tyrs are rather slow as in d-amino acid oxidase,^37^ because *E*_IP_ is higher by 0.8 eV than that of Trp.^54^ The biological significance of Tyr cation radicals produced as a consequence of ET to Iso* is not known in the flavoproteins described here.

## IV. Conclusion

Reliability of our method of ET analyses in flavoproteins is discussed in the previous work.^36^ EXDL profiles often show a parabolic behavior when ET rates are faster than *ca.* 1 ps^−1^. It is also known that SEGL shows parabolic functions with −SFEG. In the present study, we have demonstrated that EXDL with a parabolic behavior and SEGL were equivalent, showing that the values of *X*_m_(ES) obtained by SEGL were close to the values of *X*_m_(ESRc) obtained by ESRC using *X*_m_(Rc) values. It has been discussed that ET rates in the Rc-inverted region may not be trusted because the MKM theory does not hold any more, instead the phenomena should be quantum mechanically interpreted.^19,20,28,29^ However, the phenomena of the E-inverted region in SEGL have been often observed.^24–27^ The phenomena in E-inverted region do not seem to be physically unreasonable. The physical meaning of the ET rates in the Rc-inverted region should be reconsidered taking into account the equivalence between the Rc-inverted region and the E-inverted region.

## Conflicts of interest

There are no conflicts to declare.

## Supplementary Material

RA-011-D0RA09716K-s001
