# Supplementary material for: Equivalence between inverted regions of the energy gap law and inverted regions of donor–acceptor distances in photoinduced electron transfer processes in flavoproteins
Source: RSC Adv. 2021 Feb 26;11(15):8821–32. doi: 10.1039/d0ra09716k (PMC8695315; doi:10.1039/d0ra09716k)
Supplement: RA-011-D0RA09716K-s001 [file RA-011-D0RA09716K-s001.pdf]

Electronic Supplementary Material (ESI) for RSC Advances.
This journal is © The Royal Society of Chemistry 2021

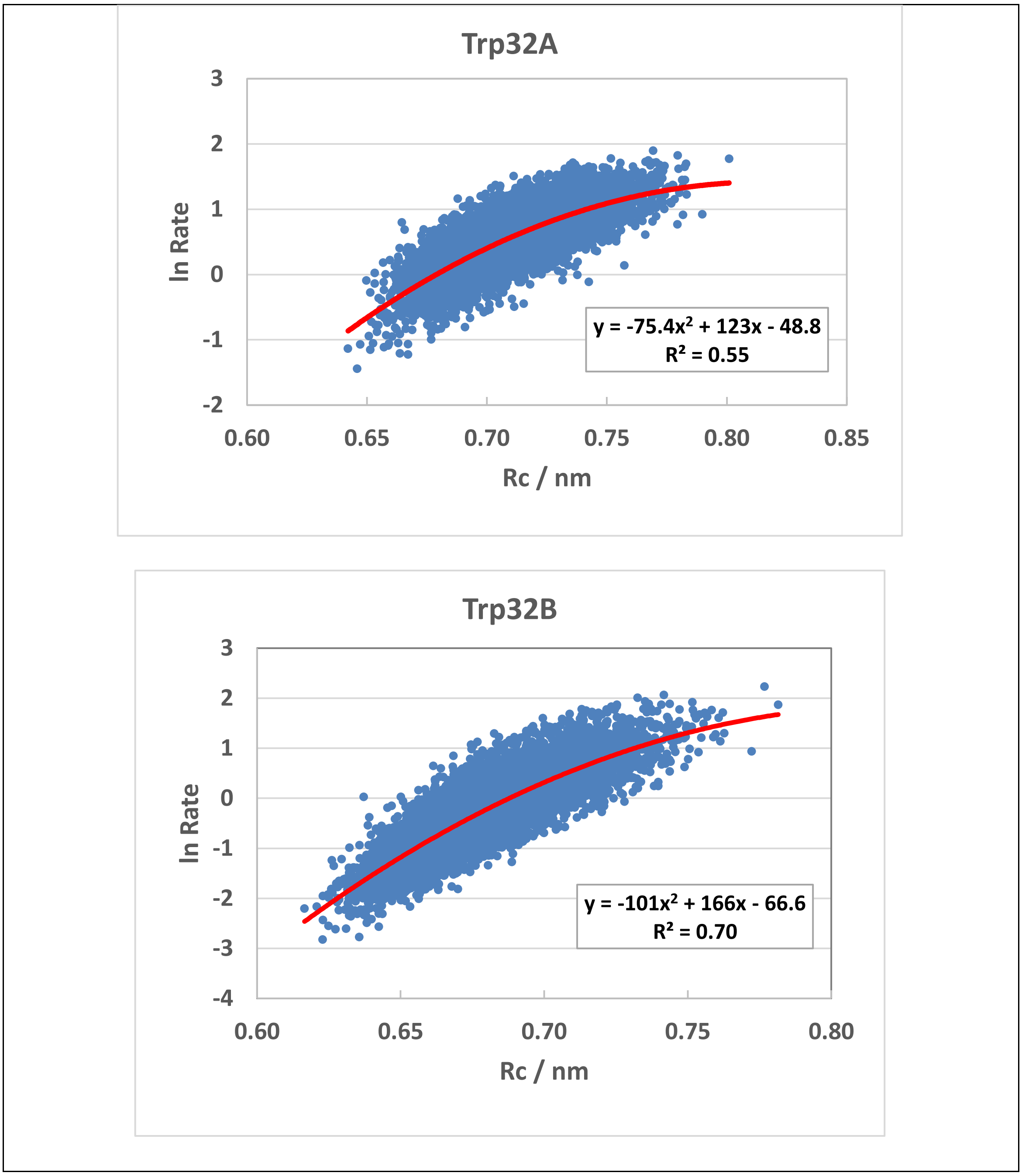

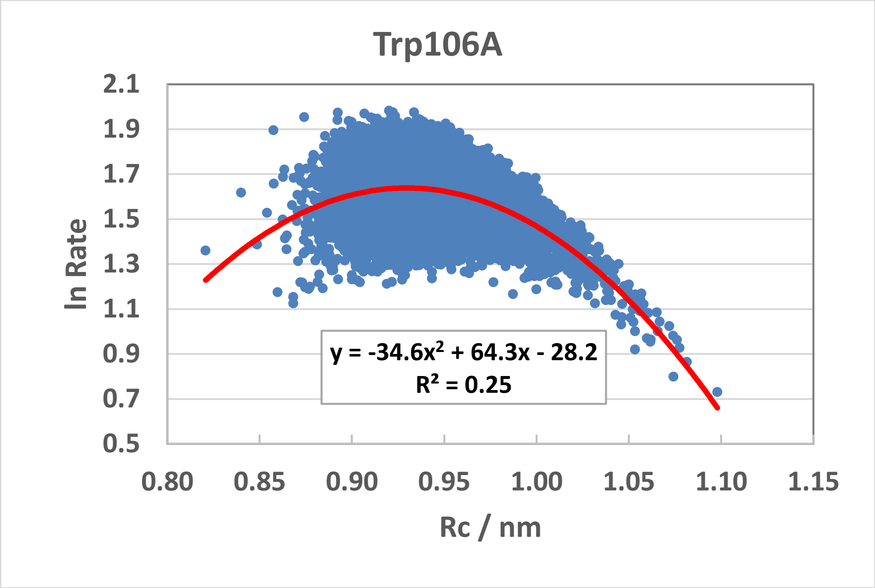

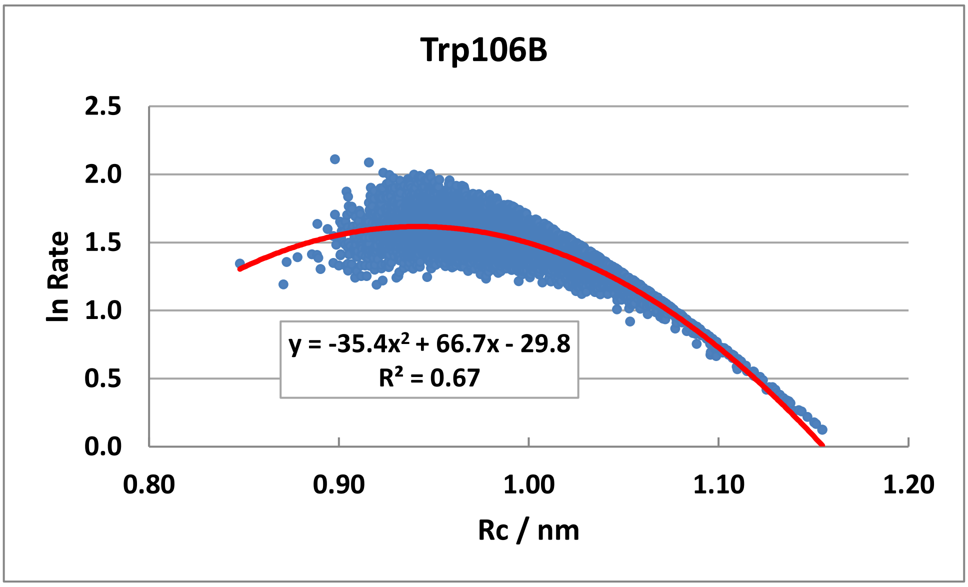

Figure S1 EXDL profiles in WT FBP[29]

Trp32A, Trp32B, Trp106A and TRp106B denote Trp32 in Sub A, Trp32 in Sub B, Trp106 in Sub A and Trp106 in Sub B as ET donors. Trp106B displayed two conformations with Rc shorter than 1.15 nm, and longer than 1.15 nm.[29] The data were collected with Rc shorter than 1.15 nm. Inserts show approximate parabolic functions. R^2 denotes determination coefficients.

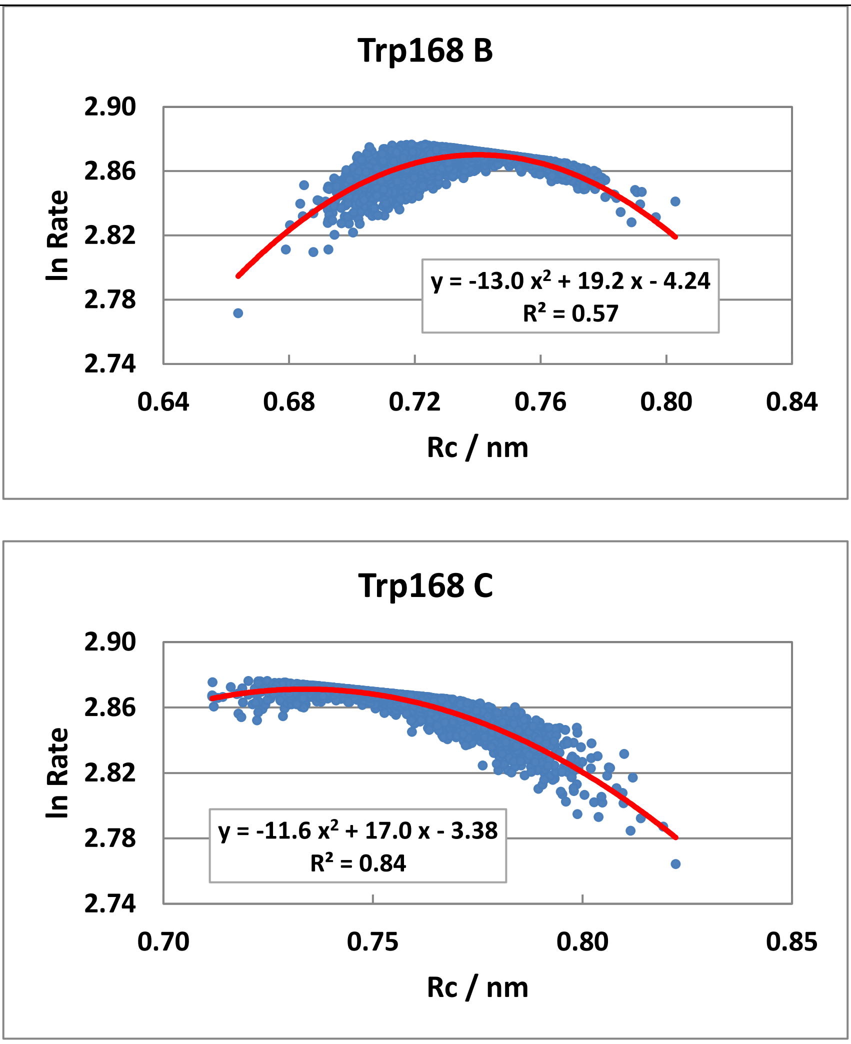

Trp168 B
2.90
2.86
2.82
2.78
2.74
ln Rate
0.64
0.68
0.72
0.76
0.80
0.84
Rc / nm
y = -13.0 x² + 19.2 x - 4.24
R² = 0.57
Trp168 C
2.90
2.86
2.82
2.78
2.74
ln Rate
0.70
0.75
0.80
0.85
Rc / nm
y = -11.6 x² + 17.0 x - 3.38
R² = 0.84

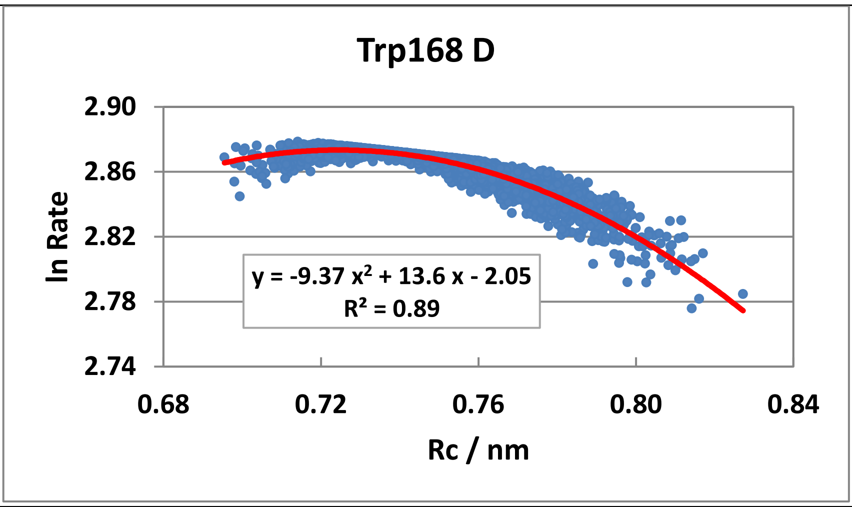

Figure S2 EXDL profiles in WT P2O[30]

Trp168B, Trp168C and Trp168D denote Trp168 in Sub A, Trp168 in Sub C, and Trp168 in Sub D as ET donors. The ET rates of these donors are fast components, which are dependent on the emission wavelength monitored for the decay measurements.[25] Emission wavelength monitored for the decay measurements is 480 nm. Inserts show approximate parabolic functions of ln Rate with Rc. R^2 denotes determination coefficients.

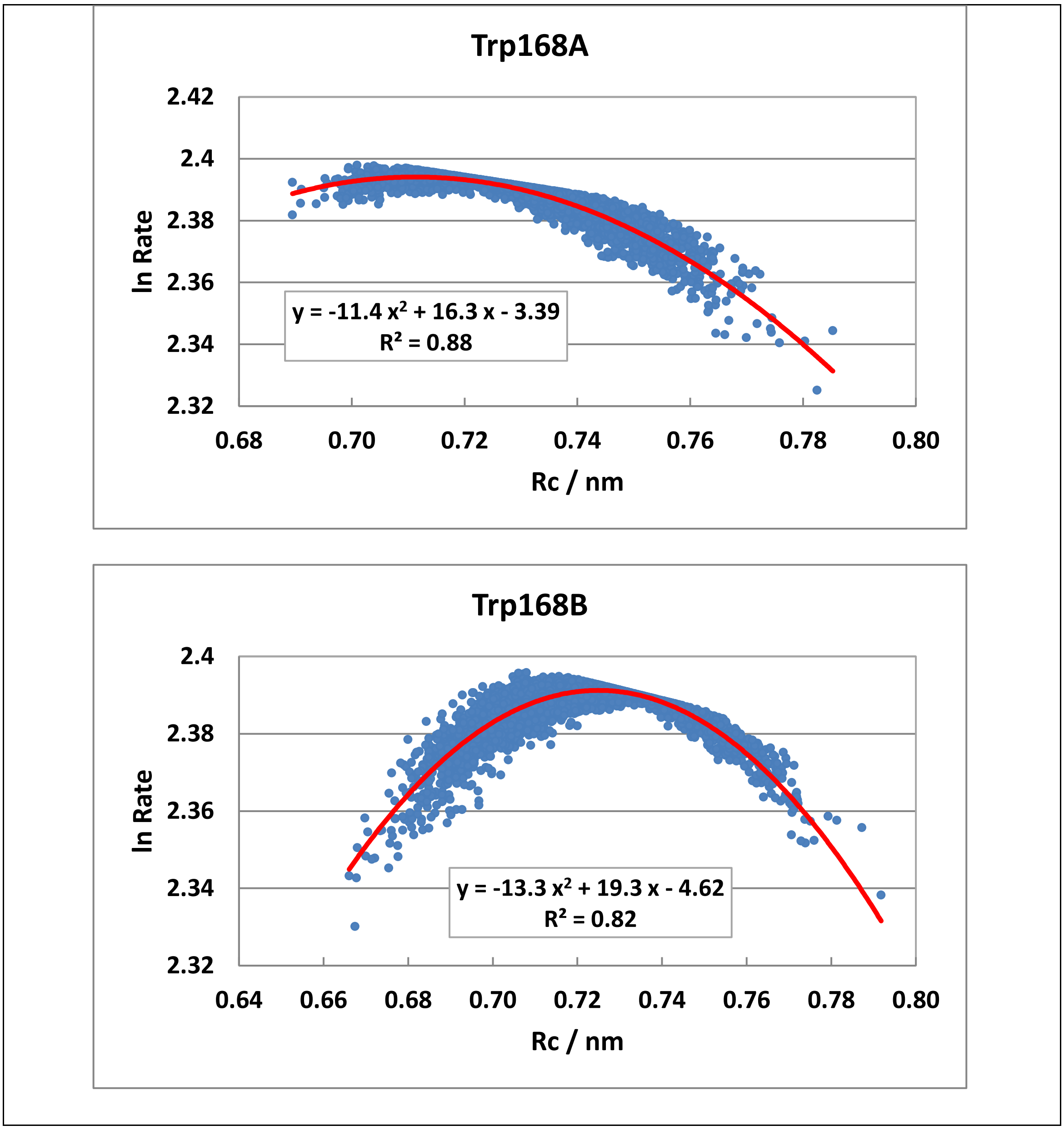
Trp168A
ln Rate
2.42
2.4
2.38
2.36
2.34
2.32
0.68
0.70
0.72
0.74
0.76
0.78
0.80
Rc / nm
y = -11.4 x² + 16.3 x - 3.39
R² = 0.88
Trp168B
ln Rate
2.4
2.38
2.36
2.34
2.32
0.64
0.66
0.68
0.70
0.72
0.74
0.76
0.78
0.80
Rc / nm
y = -13.3 x² + 19.3 x - 4.62
R² = 0.82

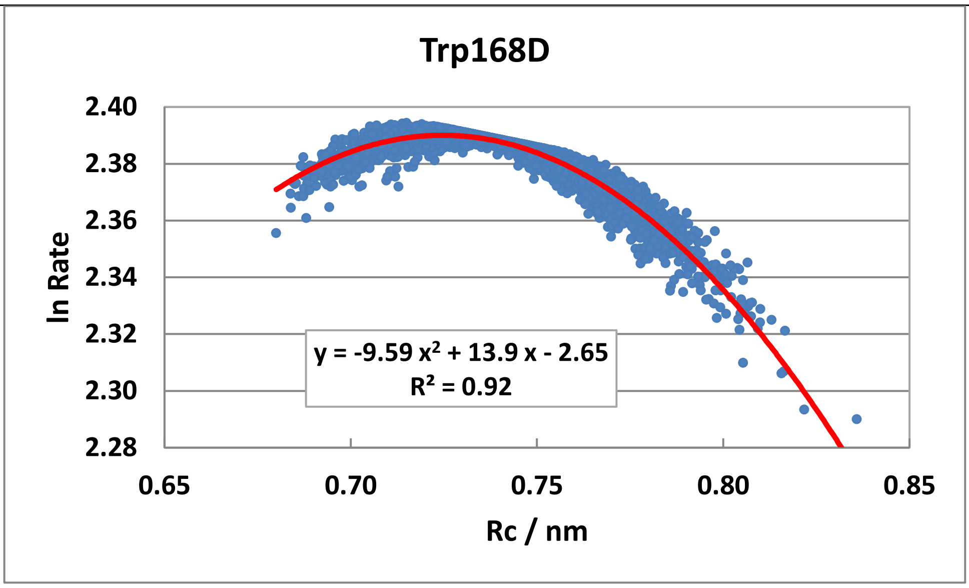

Figure S3 EXDL in T169S P2O[31]

Emission wavelength monitored for the decay measurement is 480 nm.[17] Trp168A, Trp168B, and Trp168D denote Trp168 in Sub A, in Sub B, and in Sub D respectively. The ET rates of these donors are much faster than that of Trp168C. Inserts show approximate parabolic functions of ln Rate with Rc. R^2 denotes determination coefficients.

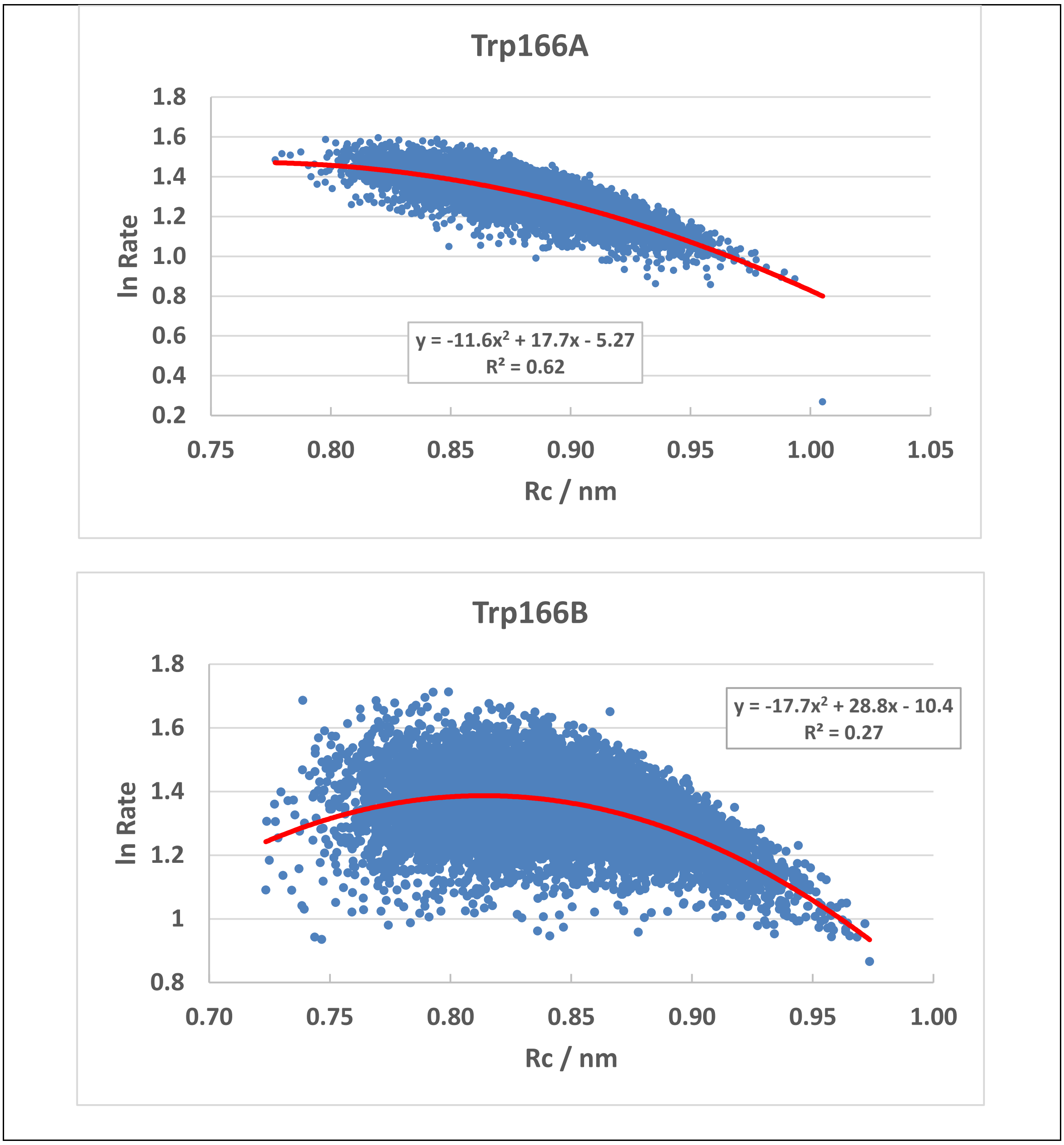
Trp166A
ln Rate
1.8
1.6
1.4
1.2
1.0
0.8
0.6
0.4
0.2
0.75
0.80
0.85
0.90
0.95
1.00
1.05
Rc / nm
y = -11.6x² + 17.7x - 5.27
R² = 0.62
Trp166B
ln Rate
1.8
1.6
1.4
1.2
1
0.8
0.70
0.75
0.80
0.85
0.90
0.95
1.00
Rc / nm
y = -17.7x² + 28.8x - 10.4
R² = 0.27

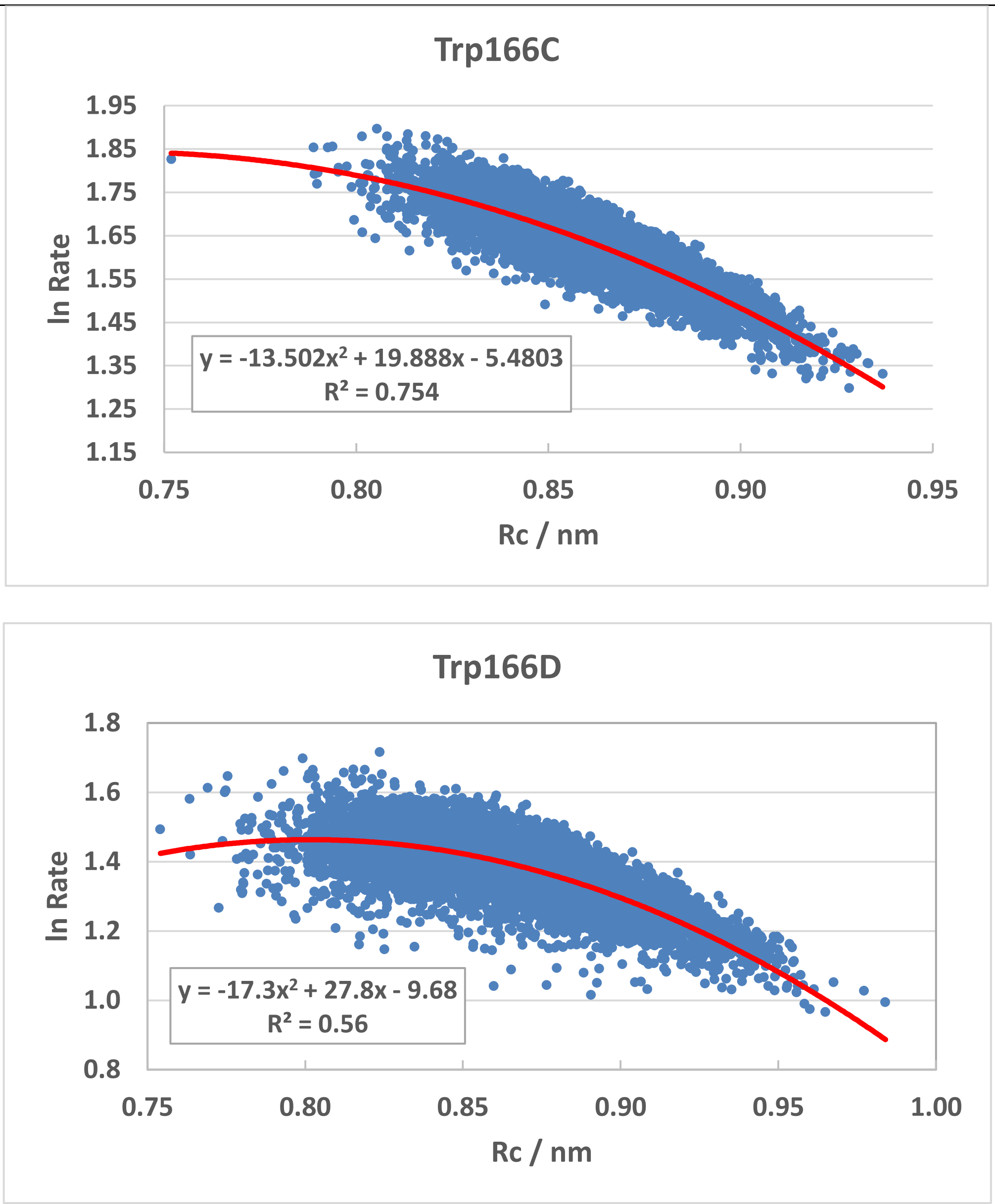

Figure S4 EXDL in MCAD[45]

The ET rates are fastest from Trp166 in any subunits among the four Trps. Trp166A, Trp166B, Trp166C and Trp166D denote Trp166 as ET donors in subunits A, B, C,and D, respectively. Inserts show approximate parabolic functions of ln Rate with Rc. R^2 denotes determination coefficient.

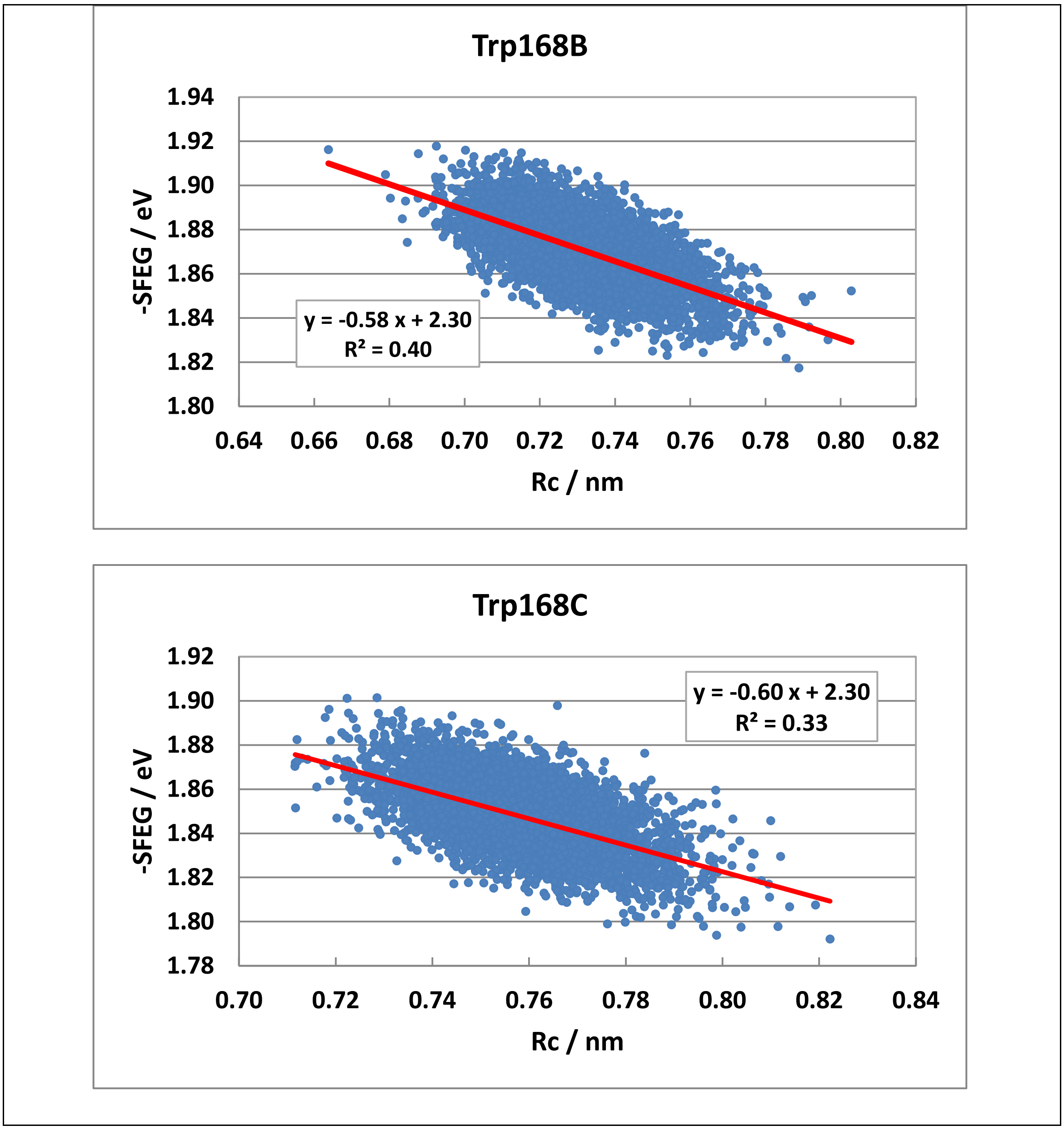
Trp168B
-SFEG / eV
1.94
1.92
1.90
1.88
1.86
1.84
1.82
1.80
0.64
0.66
0.68
0.70
0.72
0.74
0.76
0.78
0.80
0.82
Rc / nm
y = -0.58 x + 2.30
R² = 0.40
Trp168C
-SFEG / eV
1.92
1.90
1.88
1.86
1.84
1.82
1.80
1.78
0.70
0.72
0.74
0.76
0.78
0.80
0.82
0.84
Rc / nm
y = -0.60 x + 2.30
R² = 0.33

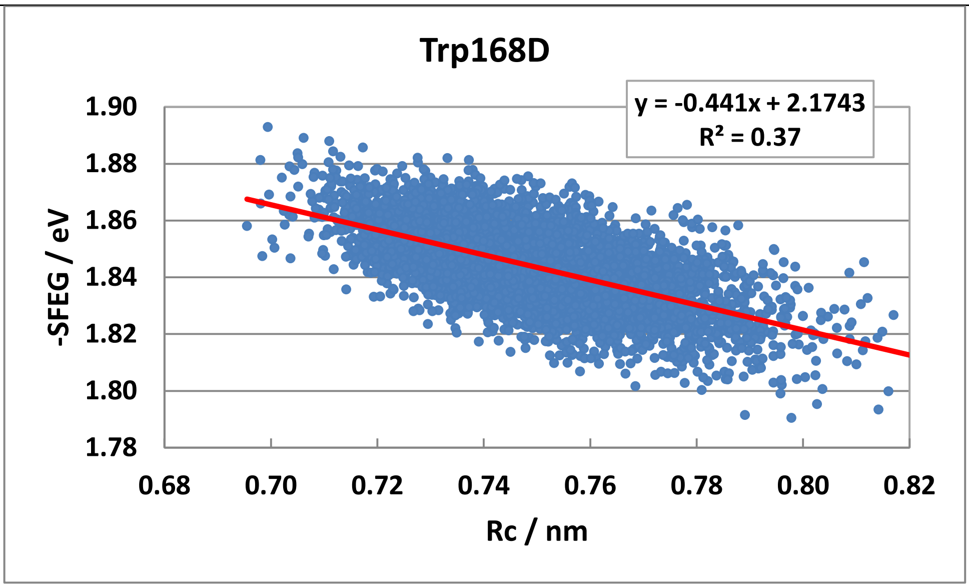

Figure S5　ESRC profiles in WT P2O

Trp168B, Trp168C and Trp168D are fast components. Emission wavelength monitored for the decay measurement is 480nm.[25] Inserts show approximate linear functions. R^2 denotes determination coefficient.

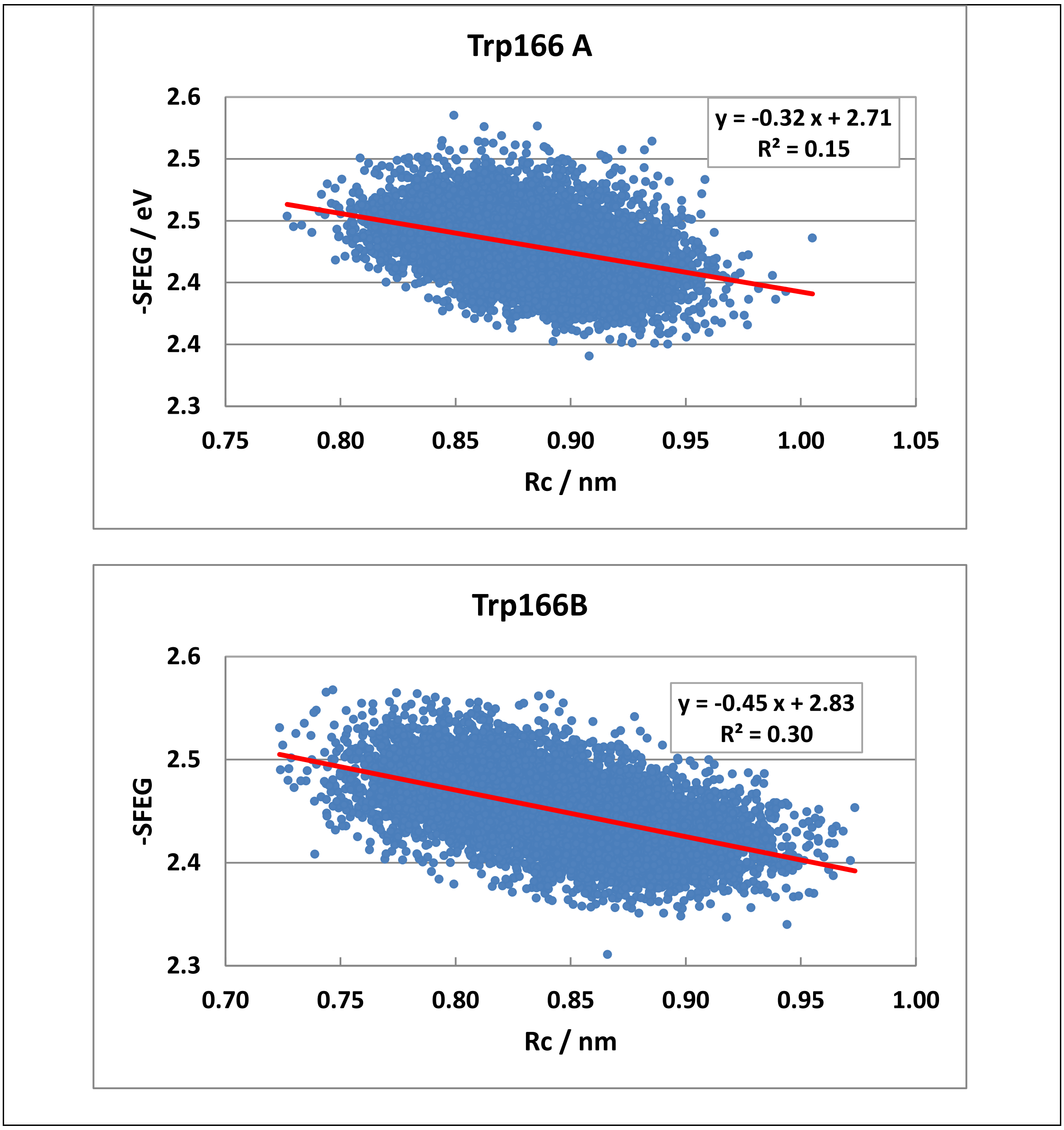
Trp166 A
y = -0.32 x + 2.71
R² = 0.15
-SFEG / eV
2.6
2.5
2.5
2.4
2.4
2.3
0.75
0.80
0.85
0.90
0.95
1.00
1.05
Rc / nm
Trp166B
y = -0.45 x + 2.83
R² = 0.30
-SFEG
2.6
2.5
2.4
2.3
0.70
0.75
0.80
0.85
0.90
0.95
1.00
Rc / nm

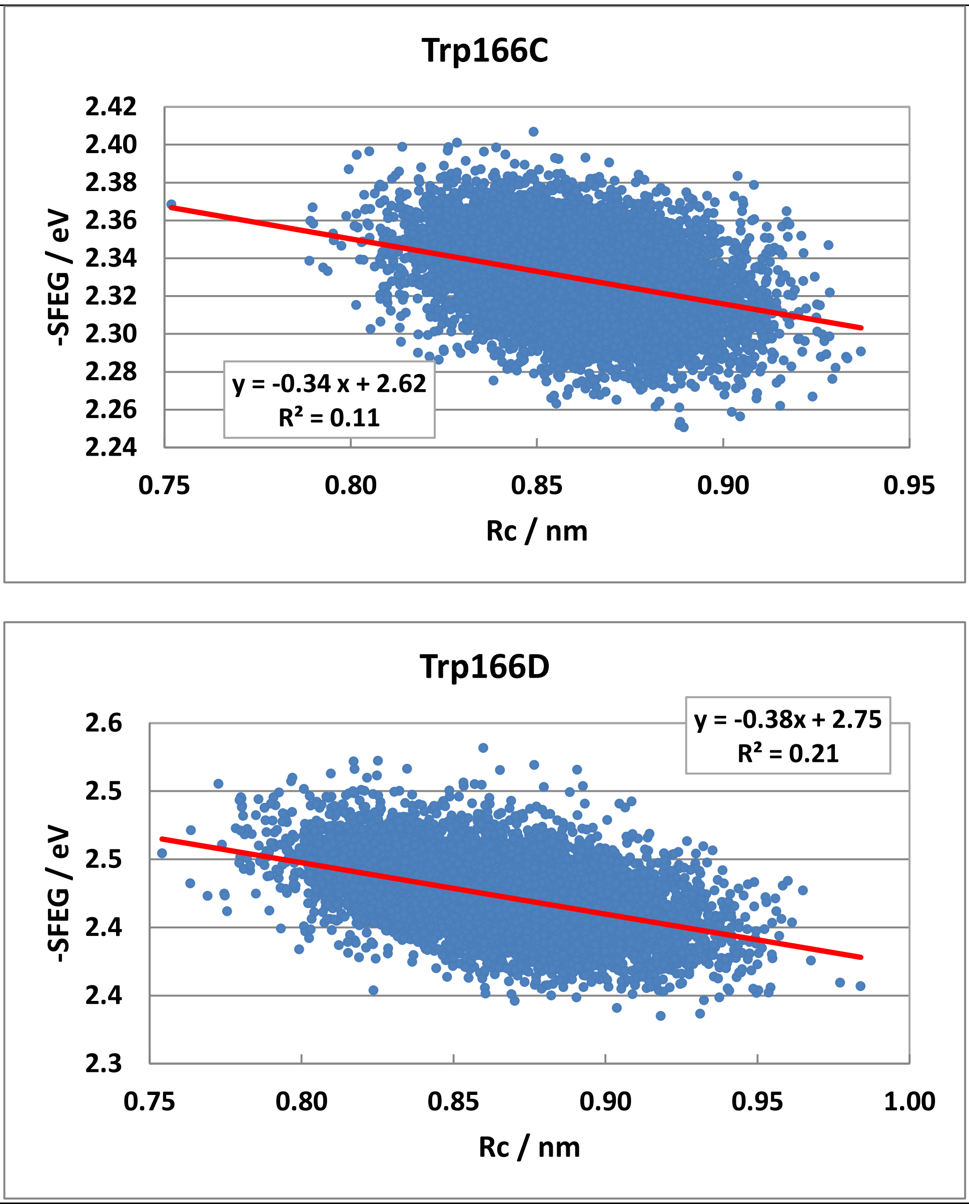

Figure S6 ESRC profiles in MCAD

Inserts show approximate linear functions. The values of SFEG are taken from the work reported.[45] R^2 denotes determination coefficient.

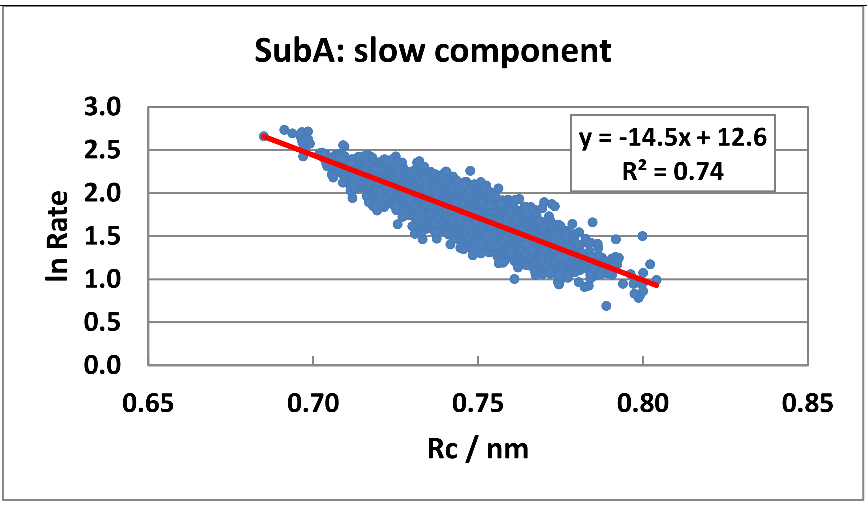
SubA: slow component
y = -14.5x + 12.6
R² = 0.74
ln Rate
Rc / nm
3.0
2.5
2.0
1.5
1.0
0.5
0.0
0.65
0.70
0.75
0.80
0.85

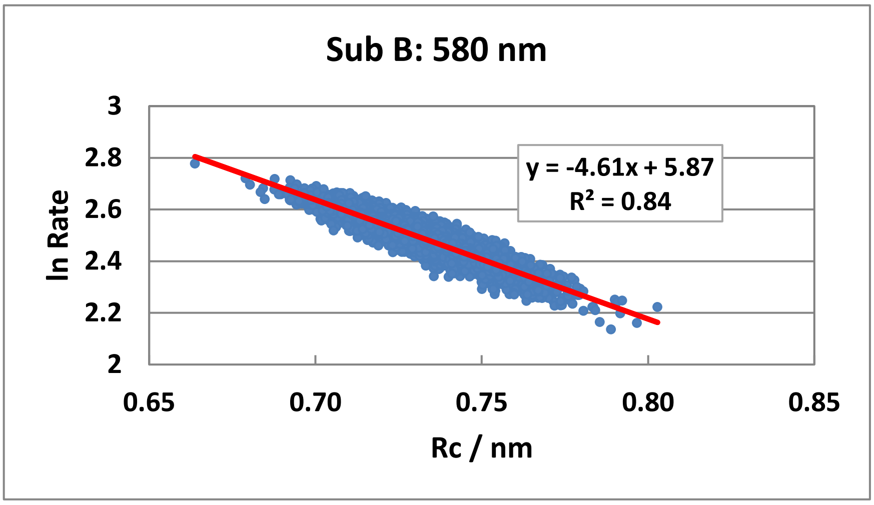
Sub B: 580 nm
y = -4.61x + 5.87
R² = 0.84
ln Rate
Rc / nm
3
2.8
2.6
2.4
2.2
2
0.65
0.70
0.75
0.80
0.85

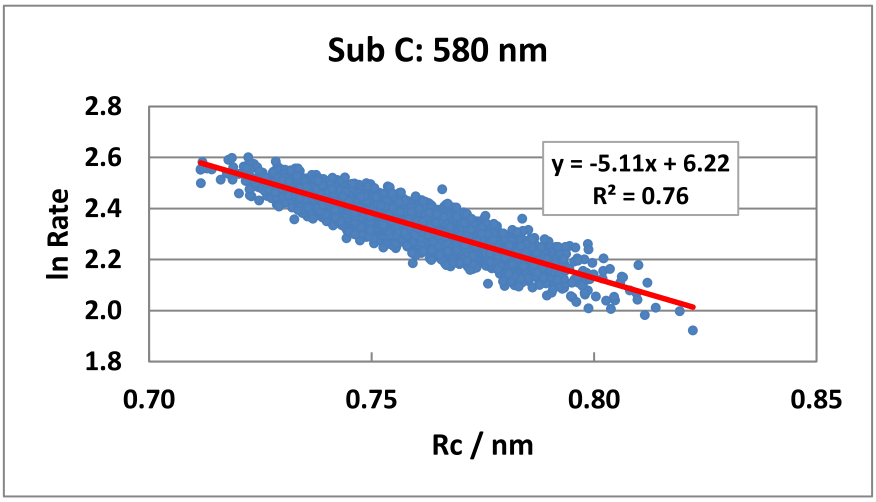
Sub C: 580 nm
y = -5.11x + 6.22
R² = 0.76
ln Rate
Rc / nm
2.8
2.6
2.4
2.2
2.0
1.8
0.70
0.75
0.80
0.85

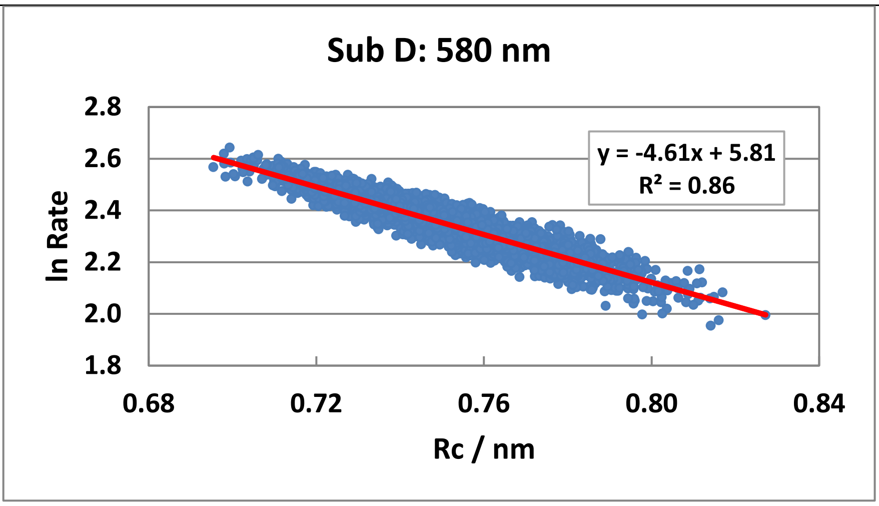

Figure S7 EXDL in WT P2O in normal region of Rc[30]

The ln Rate in the normal region linearly decreased with Rc, known as Dutton law,[32] where the rate is relatively slow. The ET rate of Trp168 in Sub A is slow component, and emission-wavelength independent.[25] The emission wavelength monitored in the fast components of Trp168B, Trp168C and Trp168D are 580 nm. Inserts shows approximate linear functions of ln Rate with Rc. R^2 denotes determination coefficient.

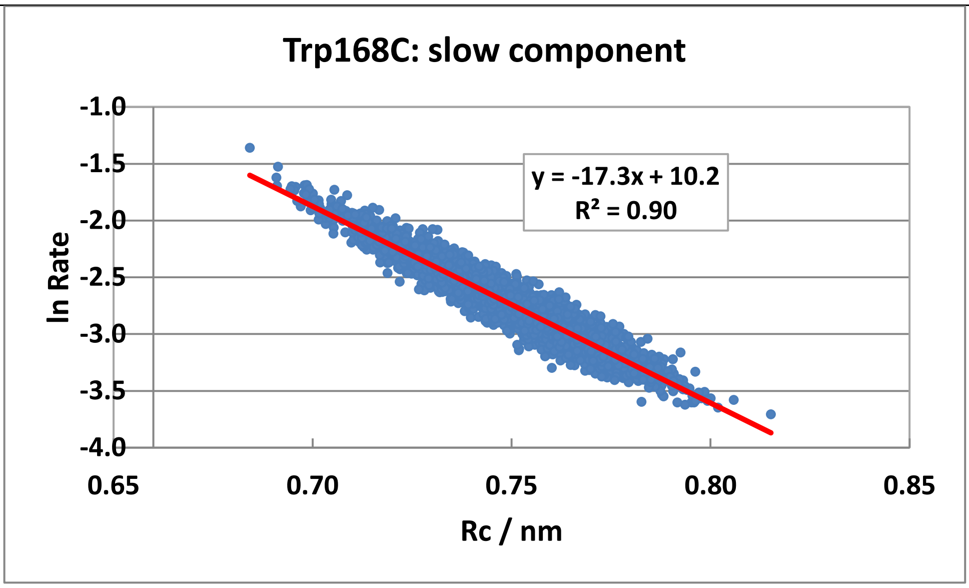
Trp168C: slow component
y = -17.3x + 10.2
R² = 0.90
ln Rate
-1.0
-1.5
-2.0
-2.5
-3.0
-3.5
-4.0
0.65
0.70
0.75
0.80
0.85
Rc / nm

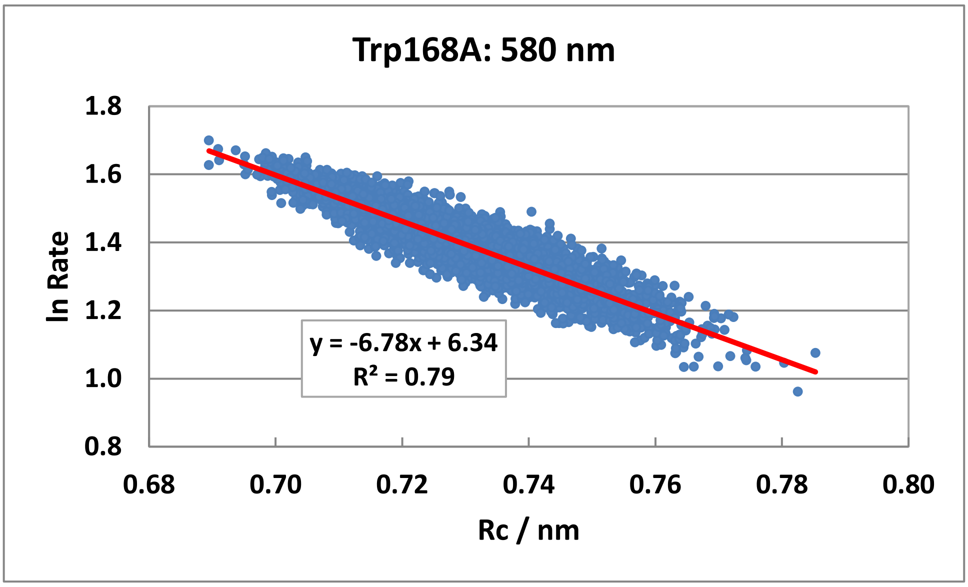
Trp168A: 580 nm
y = -6.78x + 6.34
R² = 0.79
ln Rate
1.8
1.6
1.4
1.2
1.0
0.8
0.68
0.70
0.72
0.74
0.76
0.78
0.80
Rc / nm

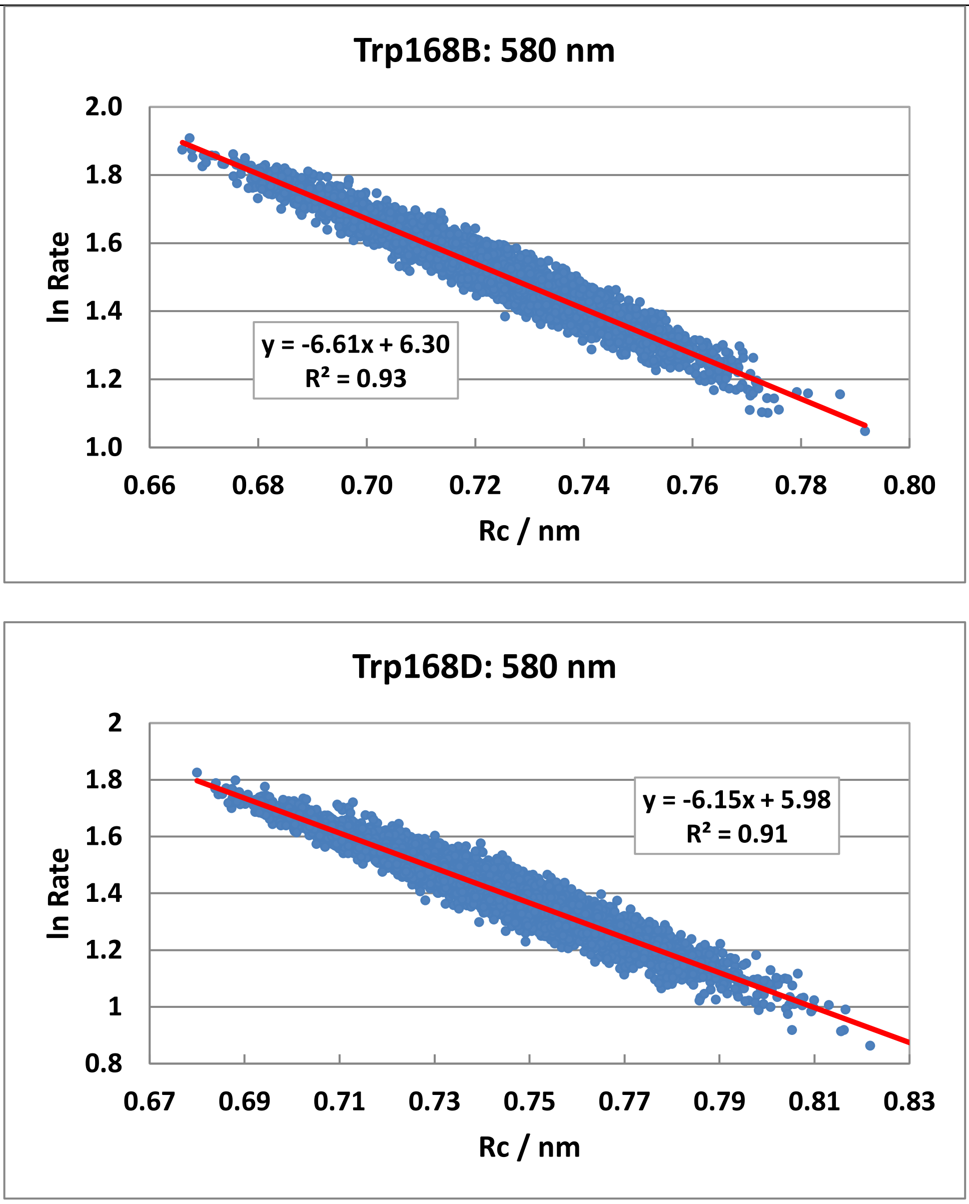

Figure S8 EXDL in T169S P2O in normal region of Rc[31]

The ln Rate in the normal region linearly decreased with Rc, known as Dutton law,[32] where the rate is relatively slow. The rate of slow component in Sub C is emission-wavelength independent.[26] The emission wavelength monitored in the fast components are 580 nm. Inserts shows approximate linear functions of ln Rate with Rc. R^2 denotes

determination coefficient.

Table S1 EXDL in flavoproteins[a]

Protein	Donor	Coefficients of parabolic function			$X_m(Rc)$ [b] (nm)	Range of Rc[c] (nm)	Reference No.
		A_1	B_1	C_1			
FBP WT	Trp32A	-75.4	123	-48.8	0.82	0.64 - 0.80	29
	Trp32B	-101	166	-66.6	0.82	0.62 - 0.78	29
	Trp106A	-35.6	64.3	-28.2	0.90	0.82 - 1.10	29
	Trp106B	-35.8	67.4	-30.2	0.94	0.82 - 1.82	29
WT P2O	Trp168B	-13	19.2	-4.24	0.74	0.66 - 0.82	30
	Trp168C	-11.6	17	-3.38	0.73	0.71 - 0.82	30
	Trp168D	-9.37	13.6	-2.04	0.73	0.69 - 0.83	30
T169S P2OT	Trp168A	-11.5	16.3	-3.4	0.71	0.68 - 0.79	31
	Trp168B	-13.3	19.3	-4.62	0.73	0.66 - 0.79	31
	Trp168D	-9.59	13.9	-2.65	0.72	0.68 - 0.84	31
MCAD	Trp166A	-11.6	17.7	-5.27	0.76	0.77 - 1.0	36
	Trp166B	-17.7	28.8	-10.4	0.81	0.72 - 0.97	45
	Trp166C	-13.5	19.9	-5.48	0.74	0.75 -0.95	45
	Trp166D	-17.3	27.8	-9.68	0.80	0.75 - 1.0	45

a The values of ln Rate were plotted against Rc, and approximated with parabolic functions, $y = A_1x^2 + B_1x + C_1$, where y = ln Rate and x = Rc.

b $X_m(Rc) = -B_1/(2A_1)$. $X_m(Rc)$ denotes x value with maximum value in y.

c Range of Rc obtained by MDS.
